# Novel semantic similarity measure improves an integrative approach to predicting gene functional associations

**DOI:** 10.1186/1752-0509-7-22

**Published:** 2013-03-14

**Authors:** Fatemeh Vafaee, Daniela Rosu, Fiona Broackes-Carter, Igor Jurisica

**Affiliations:** 1 Ontario Cancer Institute and Campbell Family Cancer Research Institute, Princess Margaret Cancer Centre, University Health Network, Toronto, Canada; 2 Department of Medical Biophysics, University of Toronto, Toronto, Canada; 3 Department of Computer Science, University of Toronto, Toronto, Canada; 4 Techna Institute, University Health Network, Toronto, Canada

**Keywords:** Gene functional association perdition, Protein interaction prediction, Functional interactome, Gene annotation, Semantic similarity measure, Systems biology

## Abstract

**Background:**

Elucidation of the direct/indirect protein interactions and gene associations is required to fully understand the workings of the cell. This can be achieved through the use of both low- and high-throughput biological experiments and *in silico* methods. We present GAP (Gene functional Association Predictor), an integrative method for predicting and characterizing gene functional associations. GAP integrates different biological features using a novel taxonomy-based semantic similarity measure in predicting and prioritizing high-quality putative gene associations. The proposed similarity measure increases information gain from the available gene annotations. The annotation information is incorporated from several public pathway databases, Gene Ontology annotations as well as drug and disease associations from the scientific literature.

**Results:**

We evaluated GAP by comparing its prediction performance with several other well-known functional interaction prediction tools over a comprehensive dataset of *known* direct and indirect interactions, and observed significantly better prediction performance. We also selected a small set of GAP’s highly-scored *novel* predicted pairs (i.e., currently not found in any known database or dataset), and by manually searching the literature for experimental evidence accessible in the public domain, we confirmed different categories of predicted functional associations with available evidence of interaction. We also provided extra supporting evidence for subset of the predicted functionally-associated pairs using an expert curated database of genes associated to autism spectrum disorders.

**Conclusions:**

GAP’s predicted “functional interactome” contains ≈1M highly-scored predicted functional associations out of which about 90% are novel (i.e., not experimentally validated). GAP’s novel predictions connect disconnected components and singletons to the main connected component of the known interactome. It can, therefore, be a valuable resource for biologists by providing corroborating evidence for and facilitating the prioritization of potential direct or indirect interactions for experimental validation. GAP is freely accessible through a web portal: http://ophid.utoronto.ca/gap.

## Background

Many different elements, e.g., DNA, protein, coding and non-coding RNA associate and cooperate to form diverse networks that make up the functional machinery of a normal living cell: the interactome. These functional associations can be defined as physical binding, such as protein-protein or protein-DNA interactions, or may refer to a group of genes or proteins involved in a signaling pathway, or encompass genetic or phenotypic associations between candidate genes in a disease process.

Our knowledge of these functional associations remains limited and fragmented. Considering protein-protein interactions (PPIs), the experimental coverage of the human proteome is at least one order of magnitude lower than the true proteome, according to some estimates [1,2], and we have even less understanding of dynamics of these interactions. Other types of interactions, such as protein-DNA or microRNA-mRNA interactions, lag behind PPIs both in coverage and curation, although there have been recent efforts to compile and integrate known interactions into databases and portals (e.g., hmChIP [3], PSICQUIC [4], mirDIP [5], and IPAD [6]).

Improving the coverage and connectivity of the interactome requires new experimental data; however, empirical methods, particularly high-throughput technologies, have a number of practical limitations and biases that cause the generation of false positives and false negatives. These can arise due to the nature and sensitivity of current methods, where, for example, stable interactions are more likely to be detected than weak or transient ones, and interactions among abundant proteins are more often detected than those with low copy number. Additionally, interactions can be condition-specific, adding yet another layer of complexity to the issue of false discovery [7,8].

*In silico* methods, despite their own limitations, can provide a complementary accompaniment to experimental methods. They are useful to conduct data quality control such as reliability assessment and validation [9,10], and they can effectively reduce noise when combining predicted interactions with experimental data [11,12]. Through an integrative analysis of biological data from different sources such as protein/DNA sequences, gene expression, and pathways, they can also facilitate knowledge exploration and interaction characterization for a deeper understanding of cellular mechanisms [13,14].

Many computational tools have focused on predicting PPI; by contrast, predicting functional associations has received less attention, with the notable exceptions of STRING [15], GeneMANIA [16] and Reactome’s FI [14]. Gene *functional association* prediction, however, has a far broader application for the interactome since it can incorporate all forms of interactions (e.g., protein-DNA, microRNA-protein, pathway co-membership) involved in a biological process, pathway or disease. Such data can be useful to elucidate cellular pathways, create functional links between genes and diseases, and can help direct and prioritize future experimental hypotheses that best fill the current gaps in our knowledge.

Our main focus in this paper is to determine whether two genes are functionally associated. We propose an *integrative* method, called Gene functional Association Predictor (GAP), which calculates a quantitative measure of gene functional relatedness, using a novel semantic similarity measure that increases information gain from the available gene annotations. The proposed semantic mechanism of inferring gene associations can process implicit evidence in order to identify and prioritize novel predictions of genes’ functional relationships.

The current version of GAP systematically integrates pathway information from multiple public online databases (e.g., Reactome, KEGG, NetPath and NCI-Nature PID), Gene Ontology annotations as well as drug and disease associations mined from PubMed. However, since the semantic similarity measure in GAP is general, it can take advantage of any type of biological data source, and can be extended to different organisms (provided that the employed databases and services cover multi-organism information). In this paper we use GAP to predict interactions among 19,027 human protein-coding genes; the gene names and symbols are those provided by the HUGO Gene Nomenclature Committee (HGNC) [17].

Several research groups have proposed methods to combine multiple information sources for computationally predicting direct and indirect gene or protein associations, e.g., [2,15,16,18-20]). These services predict likely direct and indirect associations based on conserved sequence motifs, gene fusion events, gene co-expression, orthology, and pathway co-annotation. Some of these databases also incorporate evidence obtained by text mining of the scientific literature. GAP integrates similar resources to predict level of gene association; however, its strengths comes from novel semantic similarity measure (introduced in the next section) and from using sophisticated natural language processing techniques in GoPubMed [21] to elucidate accurate information about genes from PubMed.

We have reviewed existing protein interaction prediction approaches and databases (see Additional file 1: Table S1), but selected only comparable tools for in-depth performance comparison with GAP^a^. We compared the predictive power of the selected methods with that of GAP over a gold standard dataset that we compiled from several sources of experimentally validated direct and indirect interactions. We showed that GAP has a superior *positive predictive value* (i.e., precision rate), and *specificity* vs. *sensitivity* (i.e., true positive vs. true negative rates) in identifying *known* interactions as compared to the existing interaction predictors. In support of the *novel* potential interactions predicted by GAP, we selected a set of highly-scored novel interactions (i.e., not currently found in interaction databases), and then manually searched the scientific literature for corroborating information. We also provided further supporting evidence for GAP’s predictions from an external expert-curated gene-disease association database.

## Methods

GAP calculates functional relatedness among all 19,027 human protein-coding genes using a novel taxonomy-based semantic similarity measure (as described below). This similarity measure increases information gain from the available gene annotations, and thus can be used in various subsequent analysis such as gene functional association prediction, which is the main focus of this paper.

We denote by *G* = {*g*_1_,…,*g*_*n*_} the set of *n* genes under consideration. We associate with each gene *g*_*i*_, *dfeature-sets*, $\langle F_{1}^{i},\ldots ,F_{d}^{i}\rangle$, where each feature-set $F_{k}^{i}$ includes a subset of $F_{k}=\{t_{k1},\ldots ,t_{kn_{k}}\}$, the set of all possible terms the *k*^*th*^ feature can take. For example, assume feature *F*_*GO*_ denotes all possible gene ontology terms contained in a gene ontology annotation database, and gene *g*_*i*_ is VEGFA, then the feature-set $F_{\text{GO}}^{i}$ gives the set of all GO terms in *F*_*GO*_ associated to VEGFA, e.g., $F_{\text{GO}}^{i}=\{$vascular endothelial growth factor receptor signaling pathway, foregut regionalization, lung field specification, intussusceptive angiogenesis, negative regulation of vascular permeability, trachea development, trigeminal nerve development, chromatin silencing by small RNA, chemorepellent activity, NFAT1 protein binding }.

Prior to defining a measure of similarity between two genes, we need to define similarity measures between the feature-sets corresponding to these genes. Furthermore, in order to assess the similarity between corresponding feature-sets, we need to define a strategy to evaluate the similarity between the individual terms, i.e., feature values, contained in the sets being compared.

### Term similarity measures

We use feature sets with categorical values of two types, and thus, we need two term-based similarity measures: (1) *data-driven*, for terms with no partial order over them, such as hierarchical taxonomies; and (2) *ontology-based*, for measuring the similarity between the terms whose values belong to ordered taxonomies, the Gene Ontology in our case.

#### Data-driven term similarity

We use a data-driven approach to calculate the similarity between features whose values are not part of any partially ordered structure. Even if no inherent ordering of the categorical terms is available, we can derive similarity judgements by taking advantage of supplementary information, such as the frequency of each term in the data sources included in the study. We define the similarity measure between the possible terms taken by a feature as the inverse frequency of the term, when the feature values are identical, and 0 otherwise. We define *σ*_*k*_:*F*_*k*_×*F*_*k*_↦[0,1], a similarity measure between the possible values taken by a feature *F*_*k*_, as:

(1) $$\sigma_{k}(t_{\text{ku}},t_{\text{kv}})=\left\{\begin{array}{ll} \frac{1}{f(t_{\text{ku}})} & \text{if}t_{\text{ku}}=t_{\text{kv}}\\ 0 & \text{otherwise} \end{array}\right),$$

where *f*(*t*_*ku*_) is the frequency of term *t*_*ku*_ in the data source under consideration. The intuition behind this definition is as follows: two different feature values do not contribute to the similarity of the pair of objects to which they are associated, while identical values contribute to the similarity of the pair in a manner proportional to how *informative* they are. We estimate the *informativeness* of a term as its inverse frequency in the data set under consideration. More specifically, a feature value used frequently in a data corpus is considered less informative, and has a correspondingly more modest contribution to the similarity of the pair of genes annotated with it. The same principle is applied in information retrieval techniques which rank documents based on measures built around the *inverse document frequency* of the search query terms contained in the documents to be ranked [22].

#### Ontology-based term similarity

For categorical values that belong to partially ordered sets, such as biological ontologies, we use a similarity assessment strategy based on information theoretic principles. In this approach it is assumed that the similarity between two terms is captured by their shared information, which is in turn encoded by the information in their common ancestor. Under these premises, the similarity between two terms can be estimated as the information content (IC) of their least common ancestor. In this framework, the methods for estimating the information content of a term are chosen according to the principle that the informativeness of a concept decreases with its level of abstraction, i.e., the higher in the taxonomical tree the concept is located, the lower its information content is.

There are at least two approaches for estimating the information content of an ontological concept: the *frequentist* and the *intrinsic* approach. In the *frequentist* approach, pioneered by Philip Resnik [23], the relative frequency of encountering an instance of a term in a text corpus, or database is used to quantify the information content of the term. The *intrinsic* strategy exploits the inherent structural information encoded in ontologies, which enables the modeling of the information content of ontological terms in ways that do not rely on the availability and the quality of statistical information about the usage of these terms. Intrinsic methods also subscribe to the assumption that concepts higher up in the ontology hierarchy are less informative than the concepts at lower levels, but they also make additional assumptions, such as that the number of terms a concept subsumes is proportional to how informative it is. An early example is the measure introduced by Seco and co-workers [24] for assessing the similarity of terms belonging to the WordNet [25] thesaurus.

No ontology based measure strictly dominates the others in terms of performance; thus, in GAP we implemented and evaluated multiple similarity measures, including our two novel proposals, i.e., *Leaves* and *Specificity-descendant*, described below..

The *Leaves* measure assumes that the information content of a term *t* is only proportional to the number of terminal concepts, i.e., leaf terms, subsumed by *t* in a given taxonomy. *Leaves* measure distinguishes between the most specific descendants of a term *t*, but it does not take into account the depth in the ontology of term *t* or the local density of the ontology in the neighborhood of *t*.^b^ In order to incorporate this information in the estimation of the information content of a term *t*, we introduce *Specificity-descendant* information content:

(2) $$\text{IC}(t)=f(f_{1}(\text{depth}(t)),f_{2}(\text{local\_density}(t))),$$

where *f*(*f*_1_,*f*_2_) = *f*_1_∗*f*_2_. *f*_1_ and *f*_2_ quantify the contribution of term *depth* and *local density* to the similarity assessment by taking into account all the descendants subsumed by a term *t*:

(3) $$\begin{array}{ll} \;f_{1}(\text{depth}(t)) & =\frac{\text{depth}(t)}{\underset{v\in \text{descendants}(t)}{\max}\text{depth}(v)},\\ f_{2}(\text{local\_density}(t)) & =\frac{\underset{\text{terms}}{\max}}{\text{descendants}(t)}, \end{array}$$

where *descendants*(*t*) is the number of subconcepts of *t* and max*terms* is a constant set to the number of terms in the hierarchy.

All term similarity assessment strategies have strengths and weaknesses. In particular, the information theoretic methods anchored by a frequentist approach to information content modeling are highly sensitive to (1) bias in word usage or biases in research literature, and (2) data sparseness, i.e., not all ontological concepts can be found in text corpora or annotation databases. The influence of the quality and availability of statistical data is not a factor for the performance of the intrinsic approaches for measuring the semantic similarity of ontological terms, but these measures are sensitive to the quality of the design of the ontology such as irregular levels of granularity between different sections of the ontology and the amount of overlap between the children of some concepts.

### Feature-set similarity

Estimating the similarity between a gene pair, requires calculating first the similarity between the respective feature-sets of the two comparing genes. Let *δ*_*k*_(*g*_*i*_,*g*_*j*_) be the feature-set similarity function, which assesses the similarity between $F_{k}^{i}$ and $F_{k}^{j}$, the *k*^th^ feature-sets of *g*_*i*_ and *g*_*j*_, respectively. We define our feature-set similarity function as an aggregation of the similarity scores of the terms contained in feature-sets as follows:

(4) $$\delta_{k}(g_{i},g_{j})=\frac{1}{\left|F_{k}^{i}|\times |F_{k}^{j}\right|}\underset{t_{\text{ku}}\in F_{k}^{i},t_{\text{kv}}\in F_{k}^{j}}{\sum }\sigma_{k}(t_{\text{ku}},t_{\text{kv}}),$$

and

(5) $$\delta_{k}(g_{i},g_{j})=\underset{t_{\text{ku}}\in F_{k}^{i},t_{\text{kv}}\in F_{k}^{j}}{\max}\sigma_{k}(t_{\text{ku}},t_{\text{kv}}).$$

### Gene similarity

The similarity scores for each gene feature set can be combined in several ways in order to obtain a similarity measure for pairs of genes. The *weighted sum* is a popular aggregation operator since it provides a natural way of combining the similarity assessed according to the chosen axes of comparison. We also define the similarity measure for pairs of genes as the weighted sum of the gene feature scores. We define *δ*:*G*×*G*↦R, the similarity measure for pairs of genes in *G*, as the weighted sum of the gene feature scores:

(6) $$\delta (g_{i},g_{j})=\overset{d}{\underset{k=1}{\sum }}w_{k}\delta_{k}(g_{i},g_{j}),$$

where *w*_*k*_ denotes the weight assigned to feature *F*_*k*_, and *δ*_*k*_(*g*_*i*_,*g*_*j*_) is the similarity measure associated to feature *F*_*k*_.

A weighted sum of similarity scores has the advantage of being easy to comprehend and calculate, but requires that the scores be on the same numerical scale (range). Defining a suitable technique for transforming the location and scale parameters of the similarity scores distributions is therefore essential to the meaningful aggregation of the similarity values obtained for each comparison feature.

Generally, we require the transformations of the scores to be monotonic. When the distribution of the similarity values is known, several parametric normalization approaches can be applied, e.g., the Z-score standardization for Gaussian distributions. When the distribution of the similarity scores is not known, non-parametric methods, such as quantile normalization, should be used.

Since we do not know the distribution of the similarity scores, we used two non-parametric transformation strategies for merging feature similarities into a scalar gene similarity value: (1) *decile-only* aggregation in which each feature similarity score is mapped to an integer *h*∈[1,10] such that the score is at the *h*^th^ decile of the sample, and (2) *decile-weighted* aggregation, which is a weighted sum calculation where the weights are decile values as described in decile-only method, and the scores are the (0, 1]-scaled feature similarity measures.

### The significance assessment of gene similarity scores

We estimated the statistical significance of GAP’s gene similarity scores using an empirical phenotype-based permutation test procedure that preserve the distribution of terms and feature sets’ size and structure. We randomly permuted the original feature sets among all genes, and re-computed GAP’s gene similarity scores for the permuted data. We repeated this process 10,000 times to generate a null distribution for the gene similarity scores. The statistical significance (nominal *p*-value) of the actual similarity score is then estimated relative to this null distribution (details of significance estimation are described in Additional file 1, Section 1.2).

## Materials

In this section, we give an overview of the chosen feature sources, define the gold-standard databases, and describe the performance measures that we used for evaluating GAP.

### Selected features and their source

As a general integrative framework for gene association prediction, GAP can employ any type of biological data source for extracting gene features. For the current implementation, we have extracted seven features from two major sources of scientific literature, and online biological databases. The selected features and their corresponding sources are described below, and summarized in Table 1. The last column of Table 1 reports the percentage of feature missing values, i.e., for each feature *F*_*k*_, the last column in Table 1 lists the proportion of genes *g*_*i*_’s such that $F_{k}^{i}=Ø$. About 10% of the human protein-coding genes included in this study have no feature values, according to the current version of the data sources.

**Table 1:** Information on the selected features

**Feature type**	**Feature source**	**Feature name**	**Missing feature**
			**values (%)**
	GoPubMed	GoPubMed-related-gene	24.75
		GoPubMed-related-GO	25.79
Extracted by		GoPubMed-related-disease	29.74
text-mining tools	FACTA	FACTA-related-gene	28.24
		FACTA-related-disease	32.59
		FACTA-related-drug	57.15
	Pathway databases:		
Extracted from	Reactome, KEGG,	Related-pathway	65.91
online databases	NetPath, and		
	NCI-Nature PID		

The selected features and their corresponding sources are summarized in this Table. The last column reports the percentage of feature missing values.

To keep up with the updates in the data sources integrated by GAP, we plan to update GAP quarterly. This rate of updates is computationally feasible and reasonable, as we do not expect the changes in the external data sources to notably affect GAP’s performance in shorter time intervals.

#### Text-based feature sources

We extracted text-based features from two, freely available, online scientific text search engines: FACTA and GoPubMed.

**FACTA** (Finding Associated Concepts with Text Analysis) [26]—developed by the National Center for Text Mining (NaCTeM), University of Manchester, UK—is a real-time text-mining system, which processes MEDLINE abstracts for finding and visualizing direct and indirect associations between biomedical concepts. Given a query gene, FACTA returns related biomedical concepts (e.g., genes, diseases, and drugs), and presents them in a tabular format ranked based on co-occurrence statistics. The concept IDs and their names and synonyms are collected from several biomedical databases such as UniProt, BioThesaurus, UMLS, and DrugBank.

We queried FACTA with all human protein-coding genes and stored for each query gene the set of related gene IDs^c^, UMLS and DrugBank terms.

**GoPubMed**[21]—developed by the Transinsight and the bioinformatics groups at TU Dresden, Germany—is a knowledge-based, semantic search engine that searches PubMed using Gene Ontology (GO) and Medical Subjects Headings (MeSH) terms. GoPubMed uses advanced natural language processing algorithms to provide a comprehensive and accurate semantic search. It makes use of efficient concept recognition and word sense disambiguation algorithms to cope with varying morphology, syntax and meaning of concept labels in different contexts [21]. GoPubMed also provides the search platform for GoDiseases [27], which we used in this study. GoDiseases supports functional annotation of gene products by systematically linking genes to processes, functions, diseases, etc. through evidence in the literature.

We queried GoPubMed in Jan 2012 with all human protein-coding genes and stored for each gene, the gene IDs, MeSH terms, and GO terms which were found by GoPubMed to be related to the gene of interest.

#### Online biological databases used for feature extraction

GAP also incorporates information from several curated biological pathway data-sources including Reactome-v32 [28], KEGG-v53 [29], NetPath-v1 [30], and NCI-Nature Pathway Interaction Database (NCI-PID, 2010) [31]. Pathways are an essential feature for discovering functionally interacting proteins, as proteins participating in similar pathways are presumed more likely to be co-expressed, or be involved in the same phenotype. Accordingly, using the pathway data sources, we assigned to each human gene all pathways in which its protein products are participating.

### Gold standard datasets

#### Positive examples

GAP is not designed to capture only direct protein interactions. We thus need to build up a positive gold standard consisting of both direct (i.e., physical) interactions and indirect associations (e.g., co-complex relationship and pathway co-membership). We sourced physical interactions from I2D-v1.95 (Interologous Interaction Database) [32], an on-line database of known (and predicted) mammalian and eukaryotic protein-protein interactions, which integrates interactions from several common PPI databases such as BioGrid [33], BIND [34], HPRD [35], IntAct [36], MINT [37], etc. In this paper, we used only known *human* protein interactions from I2D as the gold standard set of the positive physical interactions. Co-complex protein pairs were acquired from CORUM-v09, the Comprehensive Resource of Mammalian Protein Complexes [38]. The CORUM database is hand curated based on evidence derived from diverse experimental techniques, and it does not include high-throughput experiments. Pathway relationships are extracted from the following online databases: Reactome [28], KEGG [29], NetPath [30], and NCI-Nature Pathway Interaction Database [31].

**Figure 1:**
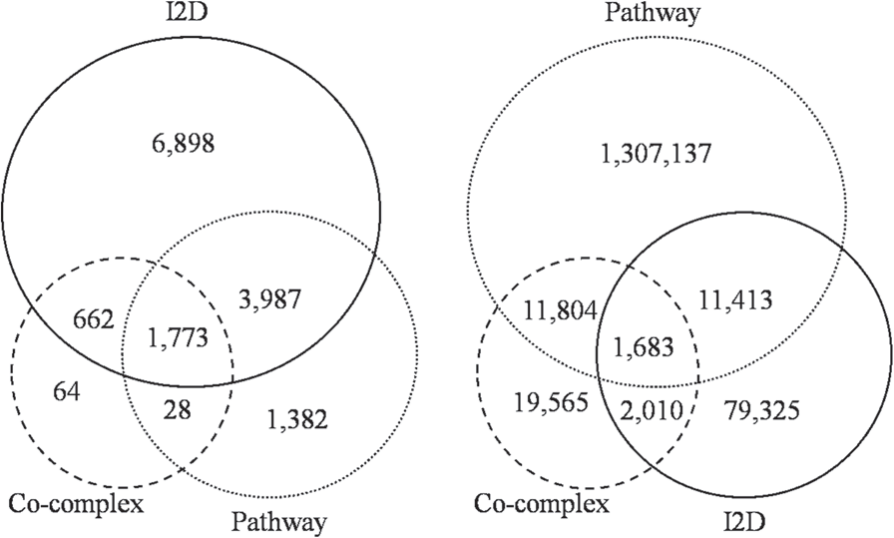
**Overlap between the three interaction data sources.** Figure 1 depicts the overlap between proteins (left) and between protein interactions (right) of the three gold standard positive databases (i.e., physical interactions, co-pathway, and co-complex associations).

Table 2 lists the number of protein pairs and the total number of proteins extracted for each of the three types of protein interactions (i.e., physical interaction, co-pathway, and co-complex). Figure 1 depicts the overlap between proteins and between protein pairs of these three sets. As Figure 1 shows, 98% of the co-pathway pairs, 56% of the co-complex pairs, and 84% of the physical interactions are distinct (i.e., do not overlap with other sets). This low overlap underscores the importance of including different types of interactions in the gold standard datasets when evaluating gene association prediction tools. We provide the set of positive examples in Additional file 2.

**Table 2:** Statistics on interaction data sources

**Type of Interaction**	**DB Statistics**	
	**No. of proteins**	**No. of protein pairs**
Physical interaction	13,320	94,431
Co-pathway	7,170	1,332,037
Co-complex	2,527	35,062
Total:	14,794	1,432,937

This Table lists the number of protein pairs and the total number of proteins extracted for each of the three types of protein interactions (i.e., physical interaction, co-pathway, and co-complex).

#### Negative examples

Unlike positive interactions, confirmed reports of non-interacting protein pairs are rarely available, especially for *indirect* interactions/associations predictors. The Negatome [39], is a collection of pairs of proteins, which are unlikely to be engaged in *direct* physical interactions. While Negatome is complementary to random datasets for training algorithms that predict direct protein interactions, it is not a suitable candidate for testing predictions of co-complex relationships or pathway co-membership. Many of the protein pairs contained in the Negatome are derived either by selecting the non-directly interacting pairs out of protein complexes with known 3D structures, or by including pairs of proteins co-occurring in biological pathways filtered against known directly interacting proteins from the IntAct database. Since many of the Negatome protein pairs are *indirectly* interacting, this dataset is unsuitable to serve as a negative sample for the purpose of this study.

The most common approach for constructing negative “gold-standards” for direct interactions is to randomly pair proteins having different cellular localization, or being involved in different biological processes [40,40,41]. Such an approach, however, is prone to an over-optimistic estimation of the predictor’s performance due to the biased selection of the negative examples, as pointed out by Ben-Hur and Noble [42]. Furthermore, restricting negative data only to pairs of proteins localized in different cellular compartments is not a good choice for evaluating predicted indirect interactions since indirectly interacting proteins that belong to the same pathway can be found in different cellular compartments (e.g., membrane-associated TGF *β* receptor and SMAD4, which can be either cytoplasmic or nuclear). Conversely, not all proteins found in the same compartment will be interacting with each other. Thus, such negative datasets are suitable for the task of predicting protein *co-localization*, rather than that of predicting direct or indirect protein interactions.

To minimize the biased distribution of negative examples, we opted for an unconstrained random sample of protein pairs, since the fraction of interacting proteins is assumed to be small compared to the total number of potential protein pairs [43]. Therefore, the possibility of including truly (unknown) interacting pairs among a random sample is low enough for our approach to yield a reasonably accurate test dataset. For each query gene, we constructed a negative set by randomly sampling gene partners from the set of available human protein-coding genes such that the size of the random set is equal to the size of the corresponding positive dataset. In general, we observed an overlap of 5% between positive and random sets. Notice that a random set is intrinsically different from a true negative set: while negative and positive sets should be logically distinct, positive and random sets may overlap regardless of the size of the sets (excluding the trivial scenario of empty positive/random sets). Excluding the overlap from the random set creates an unjustified bias to the gold standard database, and makes performance measures difficult to interpret.

### Performance evaluation measures

When queried with a gene, GAP returns a ranked list of related genes, and their corresponding association scores and evidence. The returned list of genes can be dichotomized into *related* and *unrelated* groups using any threshold-setting technique. Accordingly, GAP can be thought of as a binary classifier, and therefore, can be evaluated using typical classifier evaluation measures, which rely on a gold standard of experimentally validated direct and indirect interactions. We therefore used three common evaluation measures: F1-score [44], precision versus recall curves [44], and area under the Receiver Operator Characteristic (ROC) curve scores [45] to assess GAP performance. The interpretation of these evaluation measures in this application, and some relevant technical details are described in Additional file 1.

## Results and discussion

### GAP predictive power over the known interactions

To assess GAP’s performance over the known interactions, we randomly selected a set of 115 human genes that have some known interactions in our positive gold standard data set.^d^ GAP was then queried with these genes, one at a time, and the performance measures (e.g., F1-scores, PR curves, or AUC scores) were calculated using the score-based ranked list of the predicted interacting partners. The average of the scores, or the interpolation of the curves, were then used to measure GAP’s performance. Notice that since pathway information is included in deriving our positive gold standard dataset, to avoid the circular inclusion of the positive examples used for evaluation in the predictive feature set, the “pathway” feature is not used throughout the experiments reported in this section.

#### GAP performance at different configuration settings

We have used several methods to (1) calculate *term-based* similarities (using various data-driven and ontology-based approaches), (2) aggregate the term-based similarities into *feature* similarities (using either max and average operators), and (3) combine the feature similarities into *gene* similarity scores (using either weighted sums of feature similarity scores or *q*-quantile sums). To study the effect of each setting on GAP’s performance, we ran GAP with different configuration settings and compared the resulting performance measurements. For ease of reference, we assigned a “configuration number” to each setting as displayed in Table 3.

**Table 3:** GAP’s configuration settings

	**Configuration No.**																
		**1**	**2**	**3**	**4**	**5**	**6**	**7**	**8**	**9**	**10**	**11**	**12**	**13**	**14**	**15**	**16**
	Leaves	X	X	X	X												
Term	Resnik					X	X	X	X								
Sim.	Seco									X	X	X	X				
	SD													X	X	X	X
Feature	Avg	X	X			X	X			X	X			X	X		
Sim.	Max			X	X			X	X			X	X			X	X
Gene	Decile-w	X		X		X		X		X		X		X		X	
Sim	Decile		X		X		X		X		X		X		X		X

For the ease of reference, at any threshold setting, different combinations of term similarity, feature similarity, and gene similarity measures are given specific configuration numbers. “X” identifies the similarity measures selected for each of the configuration numbers. SD stands for *specificity-descendant* term similarity measure. Decile-w refers to *decile-weighted* gene similarity measure.

**Figure 2:**
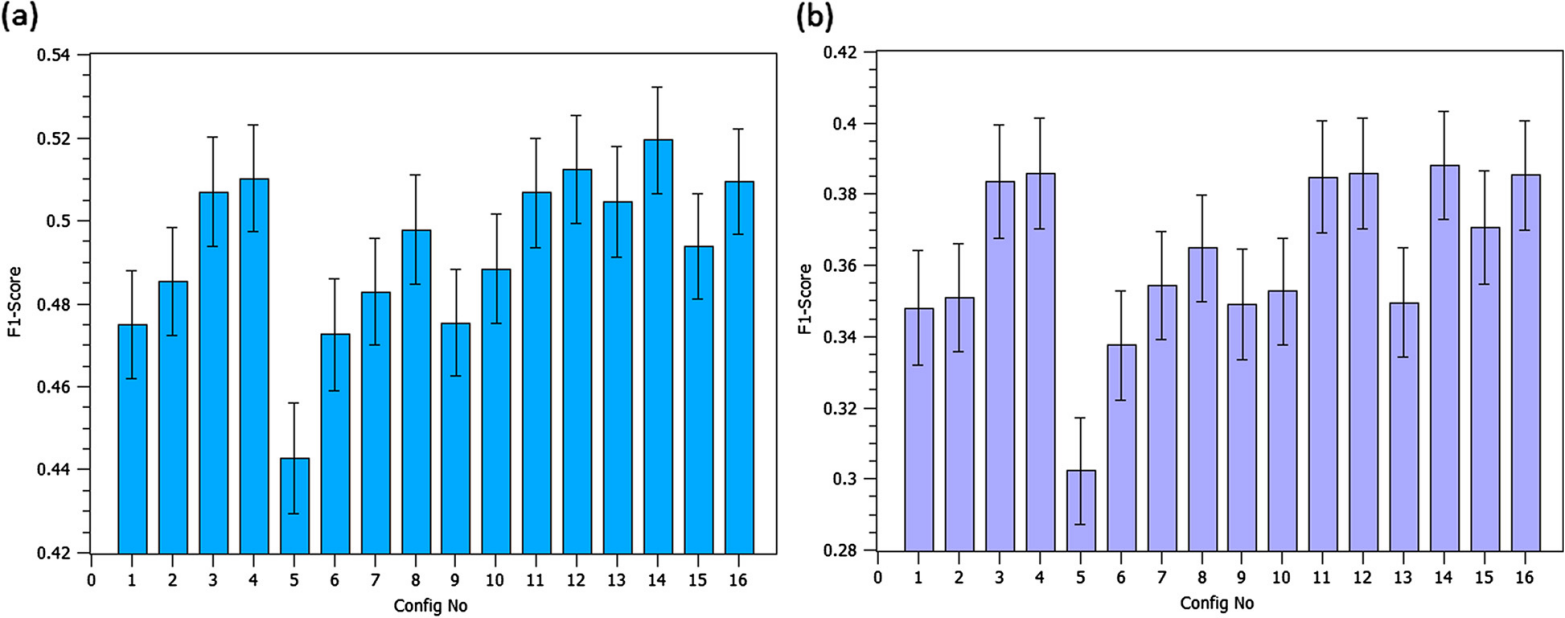
**F1-scores for different configuration settings.** F1-scores for different configuration settings when threshold is set to upper quartile **(2a)** and upper decile **(2b)**. Error bars show the standard error of the mean.

Figure 2 depicts the F1-scores for different configuration settings when the threshold is set to select the 25% and 10% of the highest-scored interactions—i.e., upper quartile and upper decile, respectively. As Figure 2 shows, the overall trend of GAP’s performance at different configuration settings is similar for both the upper quartile (Figure 2a) and upper decile (Figure 2b) threshold settings. F1-scores, however, are generally higher using the upper quartile threshold, although intuitively one may expect a decrease in performance when using a looser threshold setting, as retrieving more interactions typically introduce more noise into the prediction performance. This implies that the chance of retrieving noise (false positive) remains low while predicting more interacting pairs. Notice that the number of all possible interactions of the 115 genes with other genes in the dataset (∼19K) is 2,185,690. However, the network of known direct/indirect interactions of the selected genes, retrieved from our gold standard database, contains 6,899 unique genes and 42,228 interactions (less than 2% of all possible interactions). As this network is relatively sparse, better performance of the upper-quartile threshold setting could not be the result of a highly dense interaction network of these 115 genes in the gold standard database.

Moreover, varying gene similarity aggregation methods (decile-weighted vs. decile-only) insignificantly affects the overall performance (i.e., *p*-values >0.07 using Wilcoxon rank sum test), and therefore, no definite conclusion on the advantage of one over the other can be made, according to our experiments.

Regarding the feature similarity aggregation methods (average vs. maximum), except for when the *specificity-descendant* (SD) term similarity measure is used, maximum aggregation performs slightly better than the average aggregation. On the one hand, the average method incorporates more information from term similarities, but on the other hand, it is more prone to including noisy information, if the adopted term similarity method is not accurate enough.

Last but not least, as expected, the term similarity method used seems to be the most influential factor, as its quality would be propagated through the aggregation chain. As Figure 2 shows, GAP performs best when using SD as the term similarity measure. Configuration number 14 (SD-Avg-Decile) is the best performing configuration, and therefore, this configuration was used in GAP for all experiments, unless stated otherwise.

**Figure 3:**
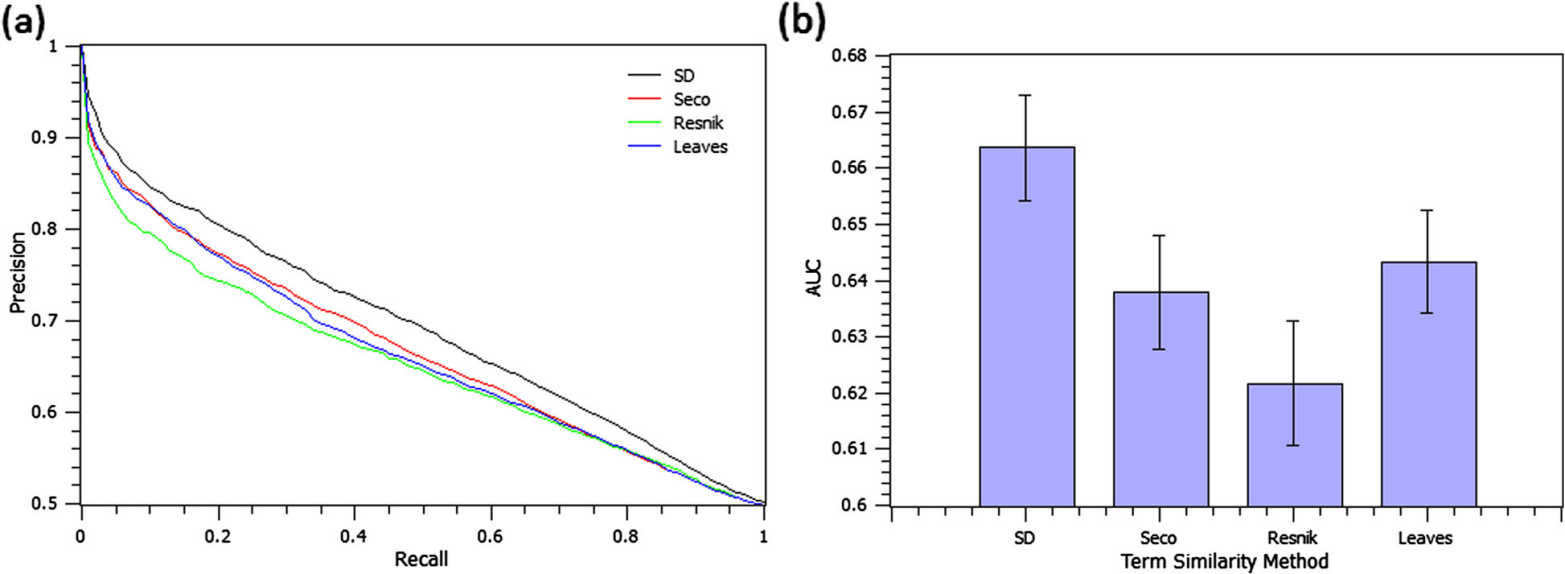
**GAP performance at different configuration settings. (3a)**: Precision versus recall curves for different term similarity measures; **(3b)**: AUC score comparison at different term similarity measures. The average of AUC values are shown as bars, and for each bar, the standard error of the mean is shown as a thin error bar.

In order to more precisely evaluate and visualize the effect of the term similarity settings, we have also reported precision versus recall curves (Figure 3a) and the AUC scores (Figure 3b) at different term similarity configurations (i.e., config. numbers 2, 6, 10, and 14). As these Figures reconfirm, SD significantly outperforms the rest of term similarity configuration settings (*p*-value <0.012 when applying Wilcoxon rank sum test on AUC scores of SD as compared to the other 3 settings).

#### GAP’s hits on positive and random datasets

**Figure 4:**
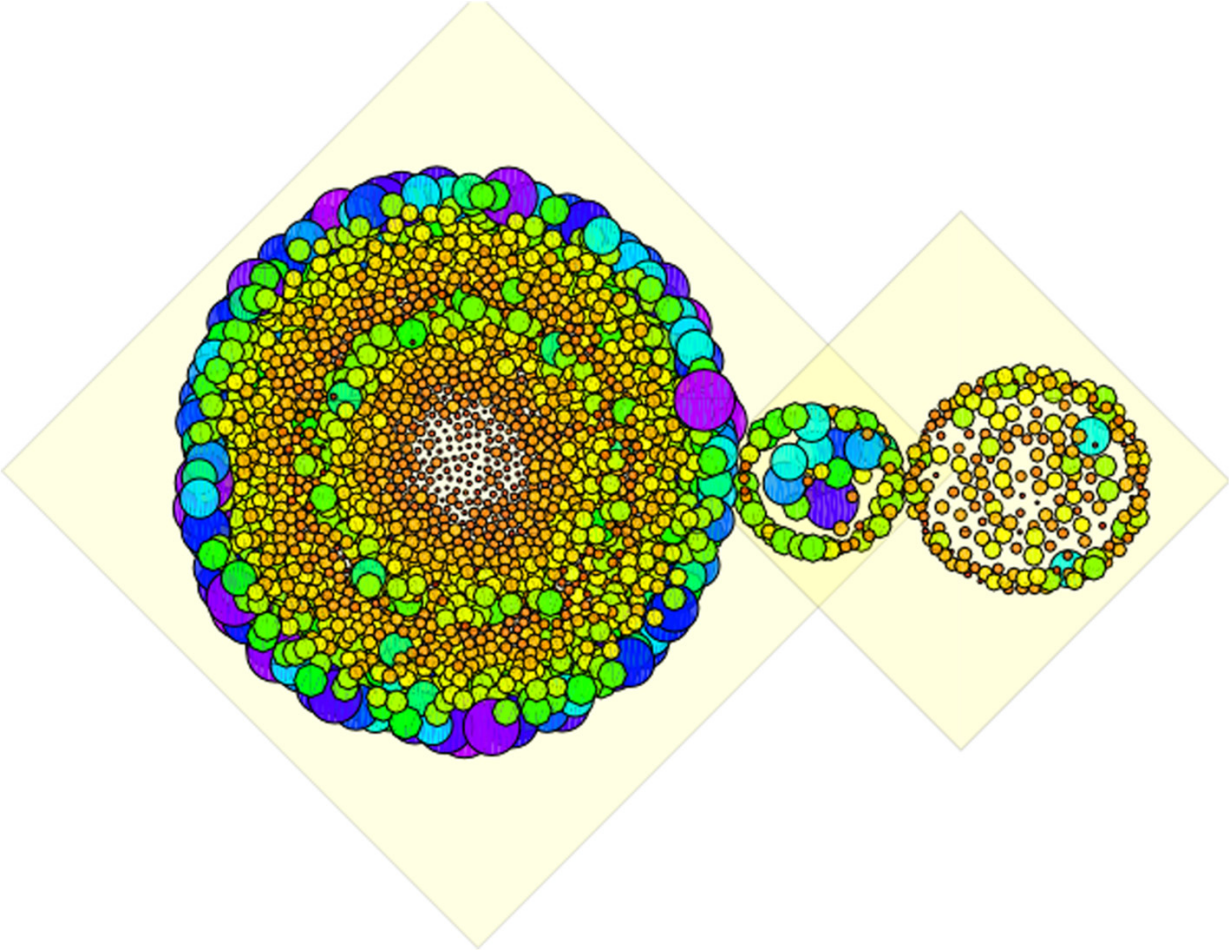
**GAP hits on positive and random datasets.** Visual representation of GAP’s hits on the positive (larger set on left) and random (smaller set on right) datasets using upper percentile threshold setting. The hits in the intersection of the two yellow diamonds are those that appear in both positive and random datasets. Each circle corresponds to an interaction predicted by GAP. The size of the circles is proportional to the score of the predicted interactions (i.e., the higher the score, the larger the size). The circles’ color changes in full spectrum from purple to red as prediction scores decrease.

Even though the overall trend of the precision vs. recall curves helps us form an impression about the rate of positive versus random hits as the scores of the predicted interactions decrease, we are interested in more precisely monitoring the proportion of positive and random hits, specifically over the highly-scored predicted interactions. We therefore collected for each of the 115 query genes considered in this study, the positive and random hits over the 10% of the highest-score predicted interactions, and displayed a set-view of the hits in Figure 4. Each circle in the Figure 4 represents an interaction predicted by GAP. The size of a circle is proportional to the score of the predicted interaction (i.e., the higher the score, the larger the size). The circles’ color changes in full spectrum from purple to red as the prediction scores decrease. Figure 4 reveals several interesting aspects of GAP’s performance. For instance, the overlap between GAP’s high-scoring predictions and the positive gold standard dataset is significantly bigger than the overlap with the random set (hypergeometric *p*-value ≈ 0.0). More precisely, the number of positive hits in Figure 4 is 2,102 while the number of negative hits is 197 (87 interactions appear in both of the positive and random sets). Furthermore, the highest-scoring interactions only appear in the positive overlap set, as the color spectrum in the overlap with the random set is more towards red with the exception of those interactions that appear in the intersection of the two sets. For instance, the proportion of negative hits among the 10% of the highest scoring predicted interactions is about 8%; this amount is reduced to 5%, and 2% if we narrow down our analysis to include only the best 5%, and 1% of the predicted interactions, respectively.

#### Predictive power of individual features

**Figure 5:**
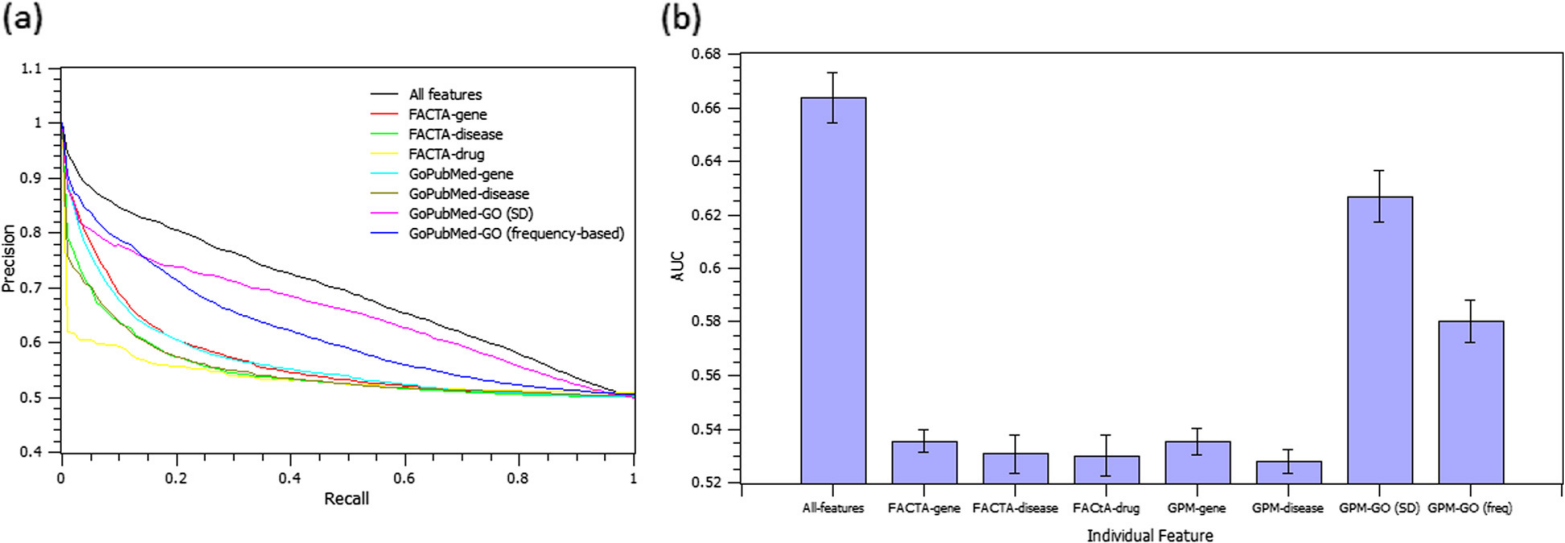
**Predictive power of individual features. (5a)**: Precision vs. recall curves using every single feature as the only predictive feature of GAP; **(5b)**: AUC score comparison when GAP is run with only one predictive feature at a time. The average of AUC values are shown as bars; the standard error of the mean is shown as a thin error bar at the top of each bar.

To assess the predictive power of each individual feature, we ran GAP with only one feature at a time, and report the interpolated PR curves and averaged AUC scores in Figures 5a and 5b, respectively. In order to investigate the advantage of using ontology annotations (the “GO” feature), we tested both ontology-based (i.e., SD) and data-driven (i.e., frequency-based) term similarity methods. As expected, the ontology-based similarity performs better, specifically at higher recall points.

As expected, combining all features together leads to the best performance (see Figure 5). Furthermore, “drug” is the least, and “GO” is the most influential feature. This observation, however, does not diminish the value of “drug” feature in deriving gene functional associations. This feature plays a part in extracting *implicit* associations, which are not sufficiently covered in our gold standard dataset. The advantage of these features would be more evident in other GAP applications such as molecular pharmacology studies rather than the protein interaction prediction.

Furthermore, it can be argued that as interaction databases influence the way gene ontologies are built, this inherent circularity would contribute to the GO predictive power. Although this circularity cannot be fully eliminated, it is minimized in GAP’s predictions as GAP makes use of primary GO annotations without any explicit access to interaction databases to infer GO term associations. The GO feature-set associated to each gene is retrieved by GoPubMed search engine by performing a knowledge-based semantic search to effectively identify ontology concepts in the texts of biomedical documents. In other words, gene ontology concepts are directly retrieved from the scientific literature using similar procedure as for other feature terms, such as disease terms.

#### GAP performance as compared to other protein interaction predictors

To ascertain GAP’s predictive power in extracting true gene associations, as compared to the existing tools, we have selected several tools, and compared them against GAP using our accumulated gold-standard dataset.

To make a fair comparison, we were interested in *functional* interaction predictors; therefore, methods specifically designed for “direct” protein interactions such as tools using molecular docking or protein structural similarity were not considered. Furthermore, we were interested in *human* gene associations, and methods particularly developed for other species were excluded. To enable the most direct comparison, we used only tools that provide web server-based prediction, or those that make the predictions available for download. Last but not least, we favored well-developed and widely-cited tools, which have been “validated” and proved useful in multiple tasks.

We applied our selection criteria to many different protein interaction predictors or gene association mining web servers, and selected (1) GeneMANIA: Gene Multiple Association Network Integration Algorithm [16], (2) I2D-Pred: Interologous Interaction Database (predicted interactions) [18], (3) PIP: Potential Interactions of Proteins [19], (4) PIPs: Human protein-protein interactions prediction database [2], (5) PPIFinder: A Mining Tool for Human Protein-Protein Interactions [20], and (6) STRING: Search Tool for the Retrieval of Interacting Genes [15].

The predictors considered for inclusion in this study, their prediction features and methodologies, and the corresponding selection constraints are listed in Additional file 1: Table S1 followed by a brief description of the six selected methods. As Table S1 shows, many computational tools have focused on predicting direct protein interactions rather than gene functional associations. Furthermore, functional interaction predictors have been more studied for other species such as worm and yeast, but received less attention for human.

**Figure 6:**
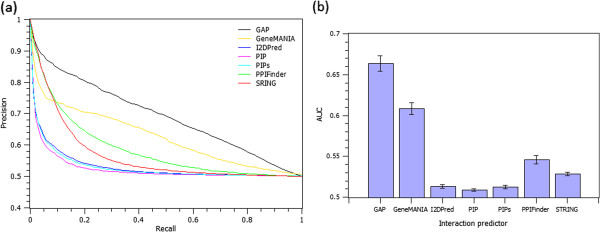
**GAP performance as compared to other predictors.** Precision vs. recall curves **(6a)**, and the average AUC scores **(6b)** of GAP and the six comparing protein interaction prediction tools. The average of AUC values are shown as bars; error bars corresponds to the standard error of the mean.

To set up the experiments, we queried each of the comparing tools with the randomly selected human genes under consideration in this study, and collected the related genes or proteins ranked according to their similarity, or confidence scores.^e^ Figure 6 shows the precision versus recall curves, and the AUC scores of GAP and the comparing protein interaction prediction tools.

The results depicted in Figure 6a and 6b suggest that GAP outperforms existing systems in extracting the experimentally known protein direct/indirect interactions (*p*-value < 2.96E-08 using Wilcoxon rank sum tests on AUC scores when comparing GAP with each of the individual tools).

### Evidence confirming and supporting GAP novel predictions

The value of protein interaction prediction and gene association mining tools lies in their ability to predict reliable novel interaction and association candidates. Biological experiments would provide validation of these predicted interactions; thus, we searched the existing literature to determine whether such supporting evidence can be identified. We also turned to manually curated databases of genes associated to high profile diseases, to support a subset of functional interactions predicted by GAP. We used, as an example, autism spectrum disorders for which there exists an expert-curated database, SFARI Gene [46].

#### Confirmation of GAP novel predictions

We queried GAP with both randomly selected genes and pre-determined genes (known metastasis suppressors), and chose for them interacting partners from the 10% of the highest-scored predicted interactions, which are also novel (i.e., protein pairs not currently found in any known database/dataset). We then searched the scientific literature for any information corroborating these predictions.

We were able to confirm functional association with available evidence of interaction for four categories of interactions: (1) direct (physical) binding, (2) interaction as part of a complex, (3) direct or indirect gene expression regulation, or (4) genetic association where genes are implicated in same/similar diseases (Table 4). Some of the confirmed interacting pairs warrant further discussion: in Dai *et al*. [47] a direct interaction between nephrin and alpha-actinin 4 was demonstrated by co-immunoprecipitation but additionally, it was shown that the nephrin-alpha-actinin 4 interaction was dependent on a third protein, integrin-linked kinase (ILK). The interaction of ILK and nephrin, either directly or as a complex, has not previously been found in any dataset/database although it is predicted by GAP to be highly associated. In all current literature, FGF2 and VEGFA belong to pathways that are treated separately, but are known to be synergistic in their action on angiogenesisi [48]; however, we were able to find a direct regulatory link between the two proteins: both appear to indirectly influence the transcription of the other’s gene. Furthermore, the known metastasis suppressor, CD82, was highly predicted by GAP to interact with AMFR. We confirmed this direct physical interaction via the literature, though it is not reported in any dataset/database. Using only known interaction data available in public database/dataset, AMFR could be connected with CD82 through shortest path of length three, our confirmation of this GAP prediction increases that to a direct connection between the two (Additional file 1: Figure S1), and thus improves the connectivity of the PPI network.

**Table 4:** Confirmation of GAP’s novel predictions

**Interactor**	**Interaction**	**Interaction**	**Confirmed by:**	**Journal**	**Year**	**Experimental**
**set**	**pair**	**type**	**Pubmed ID**			**method**
Random	ACTN4 and SYNPO	Complex	12042308	J Biol Chem	2002	Co-IP, Pull down
	FGF2 and VEGFA	Regulation	18948122	Microvasc Res	2009	N/A
	NPHS1 and ACTN4	Direct	16837631	J Am Soc Nephrol	2006	Co-IP
	VHL and SDHB	Association	19208735	Endocr Relat Cancer	2009	N/A
	BRMS1 and SAP130	Complex	16914451	Nucleic Acids Res	2006	TAP Tag
Metastasis	CD82 and AMFR	Direct	18037895	Nat Med	2007	Co-IP
Suppressor	KISS1 and KAL1	Association	22335740	N Engl J Med	2012	N/A
	KISS1 and MED23	Regulation	16964286	Oncogene	2007	ChIP

This Table summarizes a list of confirmed interactions, types of the interactions, corresponding PubMed IDs, and the experimental methods used.

#### Supporting predicted associations using an expert curated gene-disease association database

We have also found supporting evidence for our predictions by examining the Human Gene module of SFARI Gene [46], a web-based, publicly available, and expert-curated repository of genes associated with autism spectrum disorders. The content of the Human Gene module originates from published, peer-reviewed scientific literature, and is extracted through a systematic search of the literature, followed by a multi-step annotation and curation strategy.

Genes participating in the same phenotype are considered to be functionally related [49]. Since genes reported by SFARI are known to be implicated in autism spectrum disorders, we would expect to see high-density functional connectivity among the autism-related genes based on the predictions delivered by a comprehensive gene functional association predictor. We were, therefore, interested to determine how inter-connected the autism-related genes would be according to GAP’s predictions, and as compared to the existing best-performing functional predictors.

Currently, SFARI human gene module includes 306 autism-related genes (downloaded in April 2012). We report the analysis for all 306 genes in Additional file 1, but present here, for ease of visualization, an in-depth analysis of the 25 genes with the highest number of associated studies. All 25 genes were the subject of at least 10 studies, as reported by the SFARI database.

**Figure 7:**
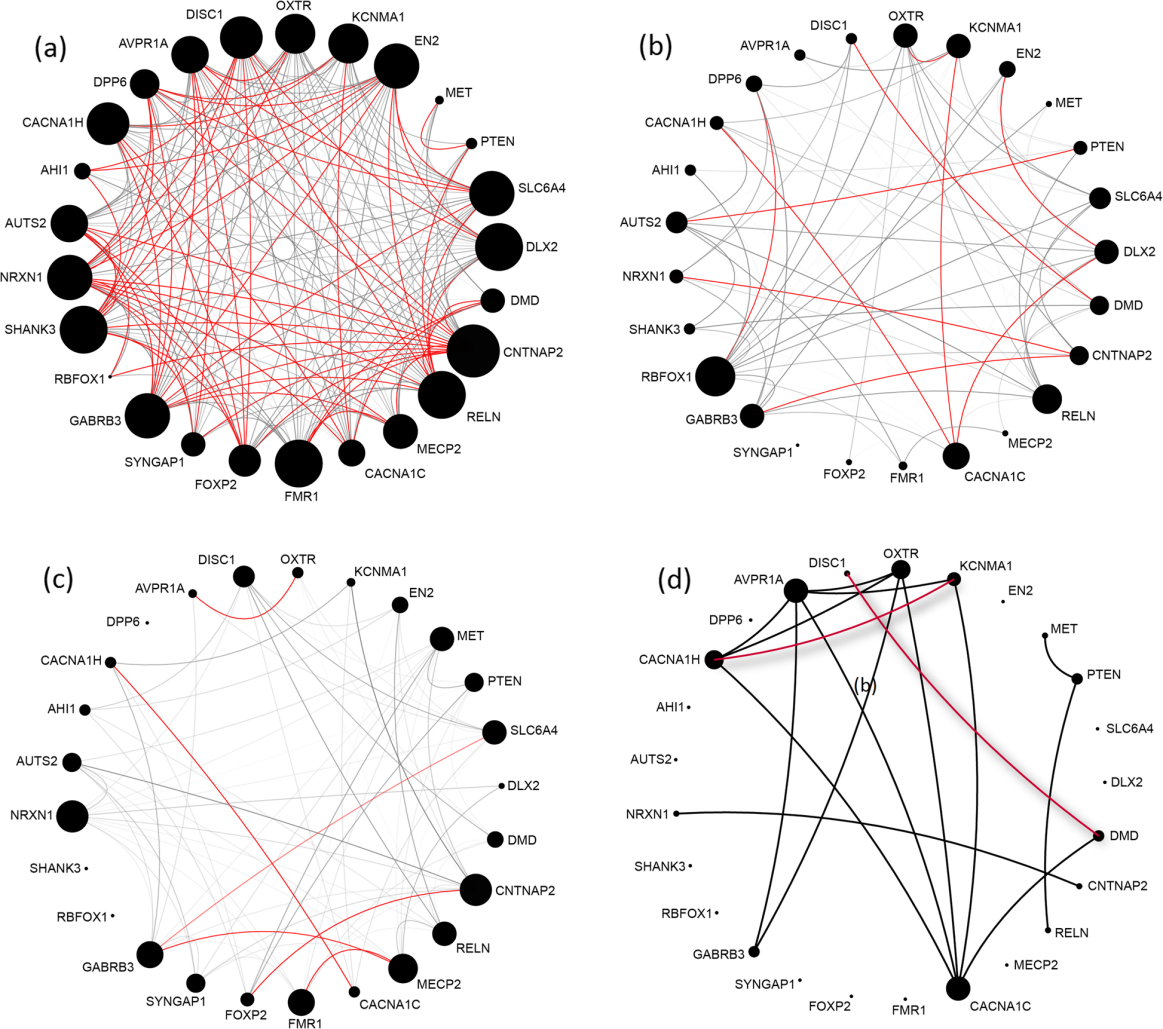
**Functional inter-connectivity of autism genes.** Functional inter-connectivity of autism genes, predicted by GAP **(7a)**, GeneMANIA **(7b)**, and STRING **(7c)**, and reported in the databases of experimentally validated direct and indirect interactions according to our gold standard dataset **(7d)**. The transparency of the edges in graphs predicted by GAP, GeneMANIA, and STRING is inversely proportional to the score of the predicted associations; the top 10% of the highest-ranked predicted interactions are colored in red. The size of each node is proportional to the node degree. In Figure 7d, red lines show direct (physical) interactions, and the rest are pathway co-membership associations.

Figure 7 displays the functional inter-connectivity of the autism-related genes predicted by GAP, as compared to GeneMANIA and STRING.^f^ The size of a node is proportional to its degree. The transparency of an edge is proportional to the relative rank of its corresponding predicted association. The top 10% highest-ranked predicted interactions are colored in red.

As Figure 7 shows, autism genes are more *densely* and more *relevantly* connected to each other, according to GAP’s predictions as compared with the other two methods. To quantitatively measure and compare the *connectivity* of the autism genes, as well as the *relevance* of the predicted connections, we used two measures of *network-centrality* and *prediction-relevance*. The network-centrality is defined as the average of the *degree centrality* of the autism genes in the networks predicted by different methods, where the *degree centrality* of a node is defined as the number of edges incident upon the node. The network-centrality measures of the GAP’s, GeneMANIA’s, and STRING’s graphs displayed in Figure 7, are 16.08, 6.16, and 6.08 respectively, where *p*-value<1.10E-08 using Wilcoxon rank sum test to assess the significance of the difference between the distributions of the degree centrality of the autism genes at GAP’s network as compared to the other two methods.

The prediction-relevance is defined as the mean value of the relative ranks of gene associations for each of the three predicted networks: GAP (61.73), GeneMANIA (39.20), and STRING (37.93). The significance of the difference in the distributions of the relative ranks for GAP’s network as compared with others was assessed using the Wilcoxon rank sum test (*p*-value<6.89E-06).

To further support GAP’s prediction selectivity, we have statistically shown that GAP does not simply predict every random pair of genes to be associated. Accordingly, we reproduced the experiment with a randomly selected set of genes: we randomly selected 25 genes, and retrieved their corresponding functional inter-connectivity network predicted by GAP. We repeated this process 50 times, and averaged the number of high-confidence predicted associations: the average numbers of edges with *p*-value ≤0.01, and ≤0.05 were 1.36, and 6.52, respectively. These statistics are respectively 22, and 88 in the network of autism-related genes (Figure 7a). This provides further evidence that GAP associates those genes that are indeed related.

Last but not least, to illustrate the utility of GAP as compared to the datasets of experimentally validated direct and indirect associations, we have also included the interactions among autism genes derived from databases of known physical interactions, complexes, and pathways using our gold standard dataset. Figure 7d shows known associations among autism genes, and exemplifies the limitation of the existing PPI and pathway databases in deriving functional associations.

#### Proposing novel candidate genes associated with autism spectrum disorders

Since GAP successfully predicted functional associations among *known* autism genes, we were encouraged to use it for predicting *novel* genes associated with autism. We queried GAP with 306 known autism genes from the SFARI human gene module, and retrieved all functionally interacting partners predicted by it whose interaction *p*-values are less than 0.05. 11,215 genes were predicted to be functionally associated to *at least* one of the SFARI-known autism genes. Notice that if we retrieve from our gold standard database, all known direct/indirect interacting partners of 306 SFARI genes, we would end up with 5,509 unique interacting genes. Considering the limited size of the currently discovered gold standard, predicting 11,215 related-genes by GAP is quite reasonable. However, as each of the SFARI genes may contribute to different processes, and participate in diverse phenotypes, not all the functionally related genes should be necessarily implicated in autism spectrum disorders. Yet, if a new gene is predicted to be functionally associated to several known autism genes, it is more likely to be involved in autism, a phenotype that is shared by all the SFARI genes.

We define the *association-degree* of a predicted gene to be the number of SFARI autism genes that are predicted by GAP to be functionally associated to it. The histogram of the association-degrees of all the 11,215 predicted novel genes is provided in Additional file 1: Figure S3. We selected predicted genes with the highest 1% of association-degrees (i.e., association-degree < 15). 114 genes were accordingly selected and reported as *novel* candidate autism genes not yet listed in the SFARI database. Figure S4 of Additional file 1 displays the network of functional associations of these 114 predicted genes to SFARI autism genes, and Additional file 3 lists for each of the 114 genes, the gene name, its interacting partners from SFARI database, and supporting evidence, i.e., pathways, GO annotations, disease and drug information used by GAP to produce the predictions.

## Conclusions

In this paper, we proposed GAP, a general-purpose integrative Gene functional Association Prediction tool. Using common performance evaluation measures and a gold-standard database of experimentally validated direct and indirect interactions, we have shown that GAP significantly outperforms the existing interaction prediction tools in correctly identifying known interactions.

While several similar systems exist, GAP uses a fundamentally different method to drive gene associations. For instance, STRING integrates and ranks gene associations by benchmarking them against a common reference dataset. In contrast to STRING, GAP follows an unsupervised strategy wherein gene associations are inferred without using any training dataset (i.e., reference database). Notice that in predicting gene functional associations, supervised methodologies undergo a serious challenge of selecting a suitable reference set: manually curated databases are accurate enough but small in size (increases the possibility of generating false negatives) and biased (literature bias), and unrestricted datasets can be large enough but inaccurate and noisy (increases the possibility of generating false positives).

GAP is also different from the unsupervised tools such as PIP, I2D, and PPI-Finder as GAP is designed to extract semantic similarities by developing a taxonomy-based scoring measure, and by using leading semantic search engines to extract association information from the published literature (e.g., GoPubMed). Other approaches mostly use direct (rather than semantic) matching (e.g., BLAST searches, homology mapping, shared GO annotations, etc.), or use co-occurrence-based and/or rule-based approaches to extract associations from PudMed abstracts (e.g., STRING searches for recurrent co-mentioning of gene names; PPI-Finder mines protein interactions based on their co-occurrences and interaction words).

Notice that GAP does not only use name co-occurrence to extract associations from the published literature; it makes use of the vast wealth of genes’ information available in the literature from the name co-occurrence and GO annotation similarity, to being associated with similar disease susceptibility and drug mechanisms. GAP therefore retrieves more information from identical data sources (e.g., scientific literature), and more precisely integrates the available information using a taxonomy-based scoring. Richer information enables GAP to select the most relevant associations, and thus to lower the possibility of generating false negatives. To decrease the chance of generating false positives, GAP weights the available data according to their “information content” and their frequencies in the scientific corpora, and then, integrates different information to prioritize stronger associations. Combining these diverse sources of information with rich semantic similarity measure ensures GAP retrieves more true positives yet with a low chance of retrieving noise (i.e., GAP achieves higher precision value at each recall point).

We have also confirmed a subset of GAP’s novel predictions, with respect to our gold standard database, by manually searching the scientific literature^g^. We then corroborated a subset of GAP’s novel predictions using an expert curated database of genes associated to the autism spectrum disorders, under the assumption that genes participating in the same phenotype are expected to be functionally related. Our primary reason for selecting autism spectrum disorders for supporting GAP’s predictions was data availability. However, autism is one of the most common neurological disorders [50] with a very complex genetic architecture whose underlying genetic determinants are still largely unknown, i.e., based on GWAS studies only a few genes with common polymorphisms have reached genome-wide significance [51]. It is, therefore, particularly useful to propose high-confidence candidate genes associated with autism susceptibility. Using GAP, we have identified 114 potential autism-related genes (cf. Additional file 3), which are not currently listed in the SFARI database.

GAP’s predicted “functional interactome”^h^ contains ≈1M predicted functional associations whose *p*-value < 0.01, out of which about 90% are novel. GAP’s novel predictions connect previously disconnected components and singletons to the main body of the known interactome. The disconnected components and singletons in the network of experimentally validated direct and indirect interactions, as well as GAP’s novel predictions are provided by Additional file 1: Figure S5.

In summary, GAP can be applied to a wide range of tasks such as phenotype prediction, gene clustering, and pharmacology analysis. The underlying approach is general, can take advantage of any type of biological data sources, and can be extended to different organisms, provided that the employed databases and services cover multi-organism information.

## Endnotes

^a^Tools that look for functional associations (i.e., not specifically designed for predicting physical interaction) among human genes, and offer a web server or make their predicted interactome available for download.

^b^Formal definitions of Leaves, Seco, and Resnik measures are provided in Additional file 1, Section 1.1.

^c^Note that the retrieved gene IDs are the genes that co-occur with the queried gene in MEDLINE abstracts. This feature provides a *transitive* evidence for gene relationship.

^d^We put this constraint to avoid selecting genes with no known interactions. All the performance measures would be 0.0 using these genes since there is no “true positive”.

^e^We used the default settings of each tool. To avoid circular reasoning, in STRING and GeneMANIA, we excluded interactions that are directly included from the databases of experimentally known interactions.

^f^Considering all compared methods for functional association prediction, GeneMANIA and STRING are the techniques that are the most comparable to GAP, as they seek gene associations in a general way. They are also among the best-performing existing predictors.

^g^Though these confirmed predictions were experimentally validated according to published literature, they are new to the publicly available datasets of known direct and indirect interactions.

^h^By “functional interactome” we mean a network in which nodes correspond to genes and edges correspond to the functional associations between the adjacent nodes.

## Competing interests

The authors declare that they have no competing interests.

## Authors’ contributions

FV designed the study and implemented the project in java. DR proposed and implemented the specificity-descendant taxonomy-based term similarity measure. Gene ontology term similarities were extracted by DR, and then incorporated to the main project by FV. FBC searched scientific literature to confirm a subset of GAP’s novel predictions, and wrote the corresponding sections. IJ supervised the project. FV wrote the initial manuscript, and all authors edited the manuscript. All authors read and approved the final manuscript.

## Supplementary Material

Additional file 1 Includes (1) detailed descriptions of the methods and materials, (2) supplementary Table S1—i.e., the list of predictors considered for inclusion in this study—followed by a brief description of the six methods selected to be compared with GAP, (3) supplementary Figure S1—i.e., the connectivity of CD82 and AMFR in the protein-protein interaction network, (4) supplementary Figure S2 and supplementary Table S2—i.e., the analysis of functional inter-connectivity of the autism-related genes predicted by GAP for all 306 genes included in SFARI dataset, (5) supplementary Figures S3 and S4—i.e., the analysis of autism-related genes predicted by GAP which are novel to the SFARI database, and (6) supplementary Figure S5—i.e., the network of GAP’s novel predictions, and the disconnected components in the network of experimentally validated direct and indirect interactions. Click here for file

Additional file 2 Includes the positive gold standard (i.e., physical interactions, co-pathway, and co-complex associations) used for the performance evaluation in this study. Click here for file

Additional file 3 Lists for each of the 114 novel autism genes predicted by GAP, the gene name, its interacting partners from SFARI database, and supporting evidence, i.e., pathways, GO annotations, disease and drug information used by GAP to produce the predictions. Click here for file

## References

[B1] Hart G How complete are current yeast and human protein-interaction networks? Genome Biol 2006 7 11 120 10.1186/gb-2006-7-11-120 17147767 PMC1794583

[B2] McDowall M PIPs: Human protein-protein interactions prediction database Nucleic Acids Res 2009 37(Database issue) D651 D656 18988626 10.1093/nar/gkn870 PMC2686497

[B3] Chen L hmChIP: a database and web server for exploring publicly available human and mouse ChIP-seq and ChIP-chip data Bioinformatics 2011 27 10 1447 1448 10.1093/bioinformatics/btr156 21450710 PMC3087956

[B4] Aranda B PSICQUIC and PSISCORE: accessing and scoring molecular interactions Nat Meth 2011 8 528 529 10.1038/nmeth.1637 PMC3246345 21716279

[B5] Shirdel E NAViGaTing the Micronome. Using multiple microrna prediction databases to identify signalling pathway-associated microRNAs PLoS ONE 2011 6 2 [10.1371/journal.pone.0017429] 10.1371/journal.pone.0017429 PMC3045450 21364759

[B6] Zhang F Drabier R IPAD: the integrated pathway analysis database for systematic enrichment analysis BMC Bioinformatics 2012 13 Suppl 15 [10.1186/1471–2105–13–S15–S7] 10.1186/1471-2105-13-S15-S7 PMC3439721 23046449

[B7] Wodak S Challenges and rewards of interaction proteomics Genome Biol 2009 8 3 18 10.1074/mcp.R800014-MCP200 18799807

[B8] Chen Y Xu D Computational analyses of high-throughput protein-protein interaction data Curr Protein Pept Sci 2003 4 3 159 181 10.2174/1389203033487225 12769716

[B9] Bader JS Gaining confidence in high-throughput protein interaction networks Nat Biotechnol 2004 22 78 85 10.1038/nbt924 14704708

[B10] Lin X Assessing reliability of protein-protein interactions by integrative analysis of data in model organisms BMC Bioinformatics 2009 29 10 Suppl 4 S5 10.1186/1471-2105-10-S4-S5 PMC2681066 19426453

[B11] You Z Using manifold embedding for assessing and predicting protein interactions from high-throughput experimental data Bioinformatics 2010 1 26(21 2744 2751 20817744 10.1093/bioinformatics/btq510 PMC3025743

[B12] Tong A A combined experimental and computational strategy to define protein interaction networks for peptide recognition modules Science 2002 295 5553 321 324 10.1126/science.1064987 11743162

[B13] Rhodes DR Probabilistic model of the human protein-protein interaction network Nat Biotechnol 2005 23 8 951 959 10.1038/nbt1103 16082366

[B14] Wu G A human functional protein interaction network and its application to cancer data analysis Genome Biol 2010 11 R53 10.1186/gb-2010-11-5-r53 20482850 PMC2898064

[B15] Szklarczyk D The STRING database in 2011: functional interaction networks of proteins, globally integrated and scored Nucleic Acids Res 2011 39 D561 d568 10.1093/nar/gkq973 21045058 PMC3013807

[B16] Mostafavi S GeneMANIA: a real-time multiple association network integration algorithm for predicting gene function Genome Biol 2008 9 S4 18613948 10.1186/gb-2008-9-s1-s4 PMC2447538

[B17] HUGO Gene Nomenclature Committee (HGNC) European Bioinformatics Institute (EMBL-EBI) Retrieved Jan,2012 from [http://www.genenames.org/]

[B18] Brown K Jurisica Unequal evolutionary conservation of human protein interactions in interologous networks Genome Biol 2007 8 5 R95 10.1186/gb-2007-8-5-r95 17535438 PMC1929159

[B19] Pall F Cluster analysis of networks generated through homology: automatic identification of important protein communities involved in cancer metastasis BMC Bioinformatics 2006 7 2 http://dx.doi.org/10.1186/1471-2105-7-2 10.1186/1471-2105-7-2 16398927 PMC1363365

[B20] He M PPI Finder: a mining tool for human protein-protein interactions PLoS ONE 2009 4 2 e4554 10.1371/journal.pone.0004554 19234603 PMC2641004

[B21] Doms A Schroeder M Semantic search with GoPubMed Semantic Tech Web, Springer 2009 5500 309 342 [http://gopubmed.org] 10.1007/978-3-642-04581-3_7

[B22] Manning CD Introduction to Information Retrieval 2008 New York: Cambridge University Press

[B23] Resnik P Using information content to evaluate semantic similarity in a taxonomy14th Int Joint Conf for AI (IJCAI-95) 1995 1 448 453

[B24] Seco N An intrinsic information content metric for semantic similarity in WordNet16th European Conf AI 2004

[B25] Miller GA WordNet: An online lexical database Int J Lexicograph 1990 3 4 235 244 10.1093/ijl/3.4.235

[B26] Tsuruoka Y FACTA: a text search engine for finding associated biomedical concepts Retrieved Jan, 2012 from [http://refine1-nactem.mc.man.ac.uk/facta/] 10.1093/bioinformatics/btn469 PMC2572701 18772154

[B27] GoDisease Powered by Transinsight Enterprise Semantic Intelligence Service Retrieved Jan, 2012 from [http://www.godisease.com/]

[B28] Matthews L Reactome knowledgebase of biological pathways and processes Nucleic Acids Res 2008 37 Database issue D619 D622 18981052 10.1093/nar/gkn863 PMC2686536

[B29] Kanehisa M Goto S KEGG: Kyoto encyclopedia of genes and genomes Nucleic Acids Res 2000 28 27 30 10.1093/nar/28.1.27 10592173 PMC102409

[B30] Kandasamy K NetPath: a public resource of curated signal transduction pathways Genome Biol 2010 11 R3 10.1186/gb-2010-11-1-r3 20067622 PMC2847715

[B31] Carl F PID: The pathway interaction database Nucleic Acids Res 2009 37 Database issue D674 D679 18832364 10.1093/nar/gkn653 PMC2686461

[B32] Brown K Jurisica I Online predicted human interaction database OPHID Bioinformatics 2005 21 9 2076 2082 10.1093/bioinformatics/bti273 15657099

[B33] Stark C BioGRID: a general repository for interaction datasets Nucleic Acids Res 2006 34 Database Issue D535 D539 16381927 10.1093/nar/gkj109 PMC1347471

[B34] Bader G Hogue C BIND–a data specification for storing and describing biomolecular interactions, molecular complexes and pathways Bioinformatics 2000 16 5 465 477 10.1093/bioinformatics/16.5.465 10871269

[B35] Peri S Development of human protein reference database as an initial platform for approaching systems biology in humans Bioinformatics 2003 13 10 2363 2371 10.1101/gr.1680803 PMC403728 14525934

[B36] Peri S The IntAct molecular interaction database in 2010 Nucleic Acids Res 2010 38 Database issue D525 D531 19850723 10.1093/nar/gkp878 PMC2808934

[B37] Zanzoni A MINT: a Molecular INTeraction database FEBS Lett 2002 513 135 140 10.1016/S0014-5793(01)03293-8 11911893

[B38] Ruepp A CORUM: the comprehensive resource of mammalian protein complexes–2009 Nucleic Acids Res 2009 38 Database issue D497 D501 19884131 10.1093/nar/gkp914 PMC2808912

[B39] Smialowski P The negatome database: a reference set of non-interacting proteinNetPath pairs Nucleic Acids Res 2010 38 Database issue D540 D544 19920129 10.1093/nar/gkp1026 PMC2808923

[B40] Jansen R A Bayesian networks approach for predicting protein-protein interactions from genomic data Science 2003 302 449 453 10.1126/science.1087361 14564010

[B41] Guo Y Using support vector machine combined with auto covariance to predict protein-protein interactions from protein sequences Nucleic Acids Res 2008 36 3025 3030 10.1093/nar/gkn159 18390576 PMC2396404

[B42] Ben-Hur A Noble WS Choosing negative examples for the prediction of protein-protein interactions BMC Bioinformatics 2007 7 Suppl 1 S2 16723005 10.1186/1471-2105-7-S1-S2 PMC1810313

[B43] Qi Y Systematic prediction of human membrane receptor interactions Proteomics 2009 9 23 5243 5255 10.1002/pmic.200900259 19798668 PMC3076061

[B44] Jones K Information retrieval experiment London:Butterworths 213 255

[B45] Fawcett T ROC graphs: notes and practical considerations for data mining researchers 2003 [Technical Report HPL-2003-4, HP Labs]

[B46] Basu S AutDB: a gene reference resource for autism research Nucleic Acids Res 2009 37(Database issue) D832 D836 [https://gene.sfari.org] 19015121 10.1093/nar/gkn835 PMC2686502

[B47] Dai C Essential role of integrin-linked kinase in podocyte biology: bridging the integrin and slit diaphragm signaling J Am Soc Nephrol 2006 17 8 2164 2175 10.1681/ASN.2006010033 16837631

[B48] Kano MR VEGF-A and FGF-2 synergistically promote neoangiogenesis through enhancement of endogenous PDGF-B–PDGFRB signaling J Cell Sci 2005 118 3759 3768 10.1242/jcs.02483 16105884

[B49] Gilman SR Rare de novo variants associated with autism implicate a large functional network of genes involved in formation and function of synapses Neuron 2011 70 5 898 907 10.1016/j.neuron.2011.05.021 21658583 PMC3607702

[B50] Geschwind DH Autism: many genes, common pathways? Cell 2008 135 391 395 10.1016/j.cell.2008.10.016 18984147 PMC2756410

[B51] Wang K Common genetic variants on 5p14.1 associate with autism spectrum disorders Nature 2009 459 528 533 10.1038/nature07999 19404256 PMC2943511

