# Supplementary material for: Novel semantic similarity measure improves an integrative approach to predicting gene functional associations
Source: BMC Syst Biol. 2013 Mar 14;7:22. doi: 10.1186/1752-0509-7-22 (PMC3663825; doi:10.1186/1752-0509-7-22)
Supplement: Additional file 1 — Includes (1) detailed descriptions of the methods and materials, (2) supplementary Table S1—i.e., the list of predictors considered for inclusion in this study—followed by a brief description of the six methods selected to be compared with GAP, (3) supplementary Figure S1—i.e., the connectivity of CD82 and AMFR in the protein-protein interaction network, (4) supplementary Figure S2 and supplementary Table S2—i.e., the analysis of functional inter-connectivity of the autism-related genes predicted by GAP for all 306 genes included in SFARI dataset, (5) supplementary Figures S3 and S4—i.e., the analysis of autism-related genes predicted by GAP which are novel to the SFARI database, and (6) supplementary Figure S5—i.e., the network of GAP’s novel predictions, and the disconnected components in the network of experimentally validated direct and indirect interactions. [file 1752-0509-7-22-S1.pdf]

Additional file 1

1 Methods

1.1 Ontology-based Term Similarity (Resnik, Seco, and Leaves Measures)

Resnik measure: In the frequentist approach, pioneered by Philip Resnik [1], the information content of an ontology term t is quantified as $-\log(p(t))$, where $p(t)$ is the probability of encountering an instance of term t in a text corpus, or database. $p(t)$ is estimated as the relative frequency $p(t) = \text{frequency}(t)/N$, where N is the total number of terms observed.

$$IC_{Resnik}(t) = -\log(p(t)). \quad (1)$$

Seco measure: An early example of intrinsic approach for estimating the information content is introduced by Seco *et al.* [2] for assessing the similarity of terms of the WordNet thesaurus:

$$IC_{Seco}(t) = \frac{\log(|hyponyms(t) + 1| / \max_t)}{\log(\frac{1}{\max_t})}, \quad (2)$$

where $hyponyms(t)$ returns the number of hyponyms of term t and $\max_t$ is a constant set to the maximum number of terms in WordNet. The denominator is a scaling factor, which ensures that the information content of the most informative term is 1.

Leaves measure: The *Leaves* measure assumes that the information content of a term t is only proportional to the number of terminal concepts, i.e., leaf terms, subsumed by t in a given taxonomy:

$$IC_{leaves}(t) = 1 - \frac{\log(|leaf\_subconcepts(t, relations) + 1|}{\log(all\_leaves(relations))}, \quad (3)$$

where $leaf\_subconcepts(t, relations)$ is the number of most specific terms subsumed by t, and $all\_leaves(relations)$ denotes all terminal terms, in the ontology graph induced by the relations under consideration, e.g., is_a and $part\_of$.

1.2 Estimating the significance of gene similarity scores

We assess the significance of the gene similarity scores, $\delta(g_i, g_j)$, returned by GAP using a phenotype-based permutation test procedure, as follows:

Let n be the number of genes, which we consider ordered, i.e., $g_1, \ldots, g_n$, and let $\pi = \{1, \ldots, n\} = \{\pi(1), \ldots, \pi(n)\}$ be a uniformly random permutation. For each feature F_k, let $\{F_k^1, \ldots, F_k^i, \ldots, F_k^n\}$ be the feature-sets associated to each of the n genes. We use π to randomly reassign these feature-sets so that F_k^i is reassigned to the $\pi(i)^{\text{th}}$ gene. We then re-compute the similarity between genes using the permuted samples. We repeat this process 10,000 times to generate a null distribution for the GAP gene similarity scores.

The nominal p-value for each $\delta(g_i, g_j)$ is then calculated as proportion of permutations samples for which the sampled gene similarity score $\delta_\pi(g_i, g_j)$ is greater than or equal to the actual gene similarity score $\delta(g_i, g_j)$:

$$p\text{-value} = \frac{1}{10,000} \sum I(\delta_\pi(g_i, g_j) \geq \delta(g_i, g_j)), \quad (4)$$

where $I(condition) = 1.0$ when the $condition$ is satisfied and 0.0 otherwise.

2 Materials

2.1 Performance Evaluation Measures

To assess GAP performance, we used F1-score, precision versus recall curves, and area under the Receiver Operator Characteristic (ROC) curve scores as described below:

2.1.1 F1-Score

F1-score [3] is a performance measure that combines precision and recall values into a single score. In our context, for each query gene, precision refers to the fraction of retrieved interacting partners that are *known* (i.e., found in the gold standard dataset) to interact with the given gene. Recall measures the fraction of the known interacting partners of the query gene that has been retrieved by the interaction prediction tool.

F1-score is the harmonic mean[1] of precision and recall, and ranges between 0 and 1. We used F1-score to compare GAP's performance for different configuration settings because the precision versus recall graph would be unreadable due to the large number of curves to be compared. Once the best performing configuration has been identified using F1-score, the precision versus recall curve is used instead for the subsequent performance comparisons.

2.1.2 Precision vs. recall curve (PR curve)

Although easy to calculate, the F1-score has certain limitations: it is not sensitive to the ranked order of the retrieved interactions as it is computed using the unordered set of retrieved interactions. As such, the rank (score) of the truly interacting protein pairs does not affect the F1-score, which is a shortcoming as, in general, users are more interested in the highly-scored predicted interactions, and expect the "true positives" to appear at the top of the ranked list of predicted interactions.

To remedy this, we used precision versus recall curves [3], which plot the precision values at every recall point. Given a query gene and a ranked list of predicted interacting partners, a PR curve is constructed by traversing down the list and plotting the precision value for each recall point. In general, a predictor A is assumed to be better than a predictor B if, at every recall point, A's precision value is higher than B's.

The precision versus recall graph is defined for a single query; however, to arrive at a meaningful conclusion, performance comparisons should be done based on several queries. We therefore, need a technique for the interpolation of precision values in order to evaluate the overall retrieval performance for a given set of queries. In this paper, we used the *ceiling* interpolation method, commonly used in the information retrieval literature [4].

2.1.3 Area Under the ROC Curve (AUC)

Receiver Operator Characteristic (ROC) curves [5] plot the true positive rate (i.e., recall) against the false-positive rate for different cutoff values of the predicted scores.[2] ROC curves, therefore, measure the tradeoff between sensitivity and specificity.

The ROC curve can be aggregated into a scalar metric by computing the area under the curve (AUC) [5]. The AUC can be interpreted as "*the expectation that a uniformly drawn random positive is ranked before a uniformly drawn random negative*" [6], which is equivalent to the Wilcoxon-Mann-Whitney U statistic test of ranks [7]. The AUC takes values between 0.0 to 1.0. Since random

[1] The harmonic mean of two numbers x_1 and x_2 can be written as $H = \frac{2x_1x_2}{x_1+x_2}$.

[2] Similar to precision versus recall curve, we used the ceiling interpolation of ROC curves corresponding to gene queries.

guessing produces the diagonal line, which has an area of 0.5, all interesting classifiers should have an AUC more than 0.5.

3 Gene association and protein interaction prediction methods compared with GAP

To comprehensively assess GAP's performance, we considered a broad range of gene association and protein-protein interaction prediction methods. We focused on methods that are functional, i.e., not specifically designed for direct protein interactions (for instance, tools using molecular docking or protein structural similarity algorithms were not selected as by design they predict only a subset of interactions). We were also favoring tools that either offer web servers or make their predicted interactome available for download. Furthermore, as we are interested in *human* gene associations, we excluded those methods specifically designed for other species.

The predictors considered for inclusion in this study, their prediction features and methodologies, and the corresponding selection constraints are listed in Table S1, which is followed by a brief description of the subset of methods selected for comparison with GAP.

Table S1: List of considered gene association or protein interaction prediction methods. Each method is referred to by the first author's name, and if available, the server's name and hyperlink is also provided. Studies are ranked chronologically. The predictive features, and the methodology used by each study is given in the second column. Constraints of each method which made us to exclude the corresponding predictor for comparison is listed in the last column.

Study/Server, if any	Features/Methodology	Selection constraints*
D. Szklarczyk et al.[8]/ `STRING`	genomic context, high-throughput experiments, co-expression, and scientific literature/ uses an integrative approach to combine protein association knowledge from different databases (a clustering-based methodology)	None
N.Tuncbag et al. [9]/ `PRISM`	structural and evolutionary information/rigid-body structural comparisons of target proteins to known template protein-protein interfaces and flexible refinement using a docking energy function	Designed for physical interaction prediction only
D. TH. Chang et al. [10]	Protein sequence and surface information/uses an approach based on relaxed variable kernel density estimator (RVKDE) for classification	Designed for physical interaction prediction only, not available
T. P. Mohamed et al. [11]	Gene ontology, co-occurrence in tissue, gene expression, sequence similarity, homology based, and domain interaction/uses four active learning algorithms for selecting the protein pairs to be used for training a random forest algorithm	Not available
I. Lee et al. [12] `WormNet`	Gene expression, physical and genetic interaction assays of *C. elegans*, scientific literature, functional associations of yeast orthologs/uses a modified Bayesian integration of different data types, and a log-likelihood scoring mechanism	Designed specifically for *C. elegans*
M. Singhal et al. [13]	Domain information/uses support vector machine algorithm	Designed for physical interaction prediction only, not available

*"Not available" means that the predicted interactome for human proteins is not available for download and there is no web server through which one can retrieve the interacting partners of the genes of interest.

Table S1 (Cont.): List of studied gene association or protein interaction prediction methods.

Study/server, if any	Features/Methodology	Selection constraints
G. Wu et al. [14]/ `Reactome-FI`	Protein-protein interactions, gene co-expression, protein domain interaction, Gene Ontology annotations and text-mined protein interactions/uses an integrative method which extends curated pathways with non-curated sources of information	Available predicted interactions are not sorted or given any score**
M. He et al. [15]/ `PPI-Finder`	PubMed abstracts and Gene Ontology annotations/mines PubMed abstract for protein co-occurrence and interaction-related keywords; searches for GO annotations, and uses the shared GO terms to infer potential PPIs	None
MD. McDowall et al. [16]/ `PIPs`	Gene expression, orthology, domain co-occurrence, post-translational modifications, co-localization, and the analysis of the local topology of the predicted PPI network/uses a naive Bayesian method that combines the feature information to derive the overall likelihood of the potential interactions	None
S. H. Park et al. [17]	Domain information/uses a knowledge driven approach, addresses pattern discovery of the interaction, and uses them for the prediction of PPI types employing Association Rule Based Classification (ARBC)	Designed for physical interaction prediction only, not available
R. Rao et al. [18]	primary sequences/uses a method based on "amino-acid residue associations" among interacting proteins	Designed for physical interaction prediction only, not available
Y. Guo et al. [19]	protein sequences/uses auto covariance and support vector machine algorithms	Predicted interactions available for yeast only
Mostafavi et al. [20]/ `GeneMANIA`	Co-expression, genetic interactions, shared protein domains, co-localization, orthology-based predictions, physical interactions, pathway reactions/uses a heuristic algorithm based on ridge regression and label progression for gene function prediction	None
J. Shen et al. [21]	Protein sequence information/uses a support vector machine algorithm combined with a kernel function and a conjoint triad feature for describing amino acids	Not available
F. Pall et al. [22]/ `PIP`	Protein sequence and homolog information/runes BLAST searches for the entire genomes of the species of interest against proteins of experimentally derived protein interaction databases; confidence scores are calculated based on the homology to experimentally observed interacting proteins and weighted according to the amount of experimental evidence available	None
A. Ben-Hur and W. S. Noble [23]	Protein sequences, gene ontology annotations, local properties of the interaction networks, and homologous interactions in other species/combines sequence-based kernels based on k-mer frequency, motif and domain content, support vector machine classifier	Predicted interactions available for yeast only

**Precision vs. recall curves and AUC scores used to evaluate methods in our study can be only applied on a *ranked* list of predicted interactions.

Table S1 (Cont.): List of studied gene association or protein interaction prediction methods.

Study/server, if any	Features/Methodology	Selection constraints
K. Brown and I. Jurisica [24]/ `I2D`	Protein sequence and orthology information/mapes model organism protein interactions to human protein orthologs using BLASTP; the predicted human interactions are those whose proteins in the model organism interaction were conserved in humans	None
XW Chen and M Liu [25]	Protein domains/uses an algorithm based on random forests of decision trees	Designed for physical interaction prediction only, predicted interactions available for yeast only
DS Han et al. [26]	Protein domain information/uses a probabilistic framework based on multiple domain combination	Designed for physical interaction prediction only, not available
P. Aloy and R. B. Russell [27]/ `InterPreTS`	protein 3D structures and homologues information/uses a heuristic search for homologues in a database of interacting domains of known 3D complex structures	Designed for physical interaction prediction only
J. R. Bock and D. A. Gough [28]	primary structure and associated physicochemical properties/ Support Vector Machine (SVM) learning system	Designed for physical interaction prediction only

3.1 Selected methods, brief description

Below is a brief description of the tools selected for comparison with GAP:

GeneMANIA: Gene Multiple Association Network Integration Algorithm [20]
The GeneMANIA algorithm comprises of : (1) a heuristic algorithm, based on ridge regression, which calculates a composite functional association network from several networks derived from different genomic or proteomic data sources, e.g., protein-protein, protein-DNA and genetic interactions, pathways, reactions, gene expression data, protein domains and phenotypic screening profiles, and (2) an efficient implementation of Gaussian field label propagation algorithms, which predict gene function given the composite network constructed by the heuristic algorithm.

I2D-Pred: Interologous Interaction Database-Predicted [24]
I2D (Interologous Interaction Database) is an on-line database of known and predicted mammalian and eukaryote protein-protein interactions. We used I2D's known human PPIs in the construction of our gold standard, and compared GAP's performance against I2D-Pred, I2D's set of 59,373 interolog-based predicted interactions.

I2D-Pred is constructed by mapping model organism (i.e., *S. cerevisiae*, *C. elegans*, *D. melanogaster*, *M. musculus*, and rat) protein interactions to human protein orthologs using BLASTP and the reciprocal best-hit approach. Using the constructed database of model organism-to-human orthologs, each model organism protein was translated to its human ortholog, and a predicted human interaction was added to the database if both proteins in the model organism interaction were conserved in humans.

PIP: Potential Interactions of Proteins [22]
PIP is a web server delivering human, rat, and fission yeast predicted protein interactions. The predictions are made via homology with experimentally derived protein-protein interactions from various species. The homologous interacting pairs of experimentally supported pritein interactions are identified by running BLAST searches for the entire genomes of the species of interest against

all proteins in the DIP [29] and MIPS [30] databases. The putative protein interactions are given confidence scores based on their homology to experimentally observed interacting proteins. The confidence scores are then weighted according to the amount of available experimental evidence, i.e., higher weight is given to more frequently observed interactions. Once the network of interacting proteins is constructed, the number of individual interactions is reduced by using a clustering method aimed at identifying key interconnected network nodes.

PIPs: Human protein-protein interactions prediction database [16]
The PIPs database is a web resource which predicts human protein-protein interactions using a nave Bayesian method that combines information from gene expression, orthology, domain co-occurrence, post-translational modifications, co-localization, and the analysis of the local topology of the predicted PPI network.

Each evidence type is considered as a separate module providing an interaction score. The individual module scores are combined into a prediction score corresponding to the overall likelihood of the potential interaction given the available data. PIPs contains 37,606 high probability interactions (i.e., with a score ≥ 1 indicating that the interaction is more likely to occur than not). Out of these, 3,400 are not reported in the HPRD, BIND, or DIP interaction databases [16].

PPI Finder: A Mining Tool for Human Protein-Protein Interactions [15]
PPIFinder is a web-based tool which mines human protein-protein interactions from PubMed abstracts based on name co-occurrence and interaction-related keywords. PPIFinder uses a hybrid frame-based approach which incorporates both statistical and computational methods. It follows a typical frequency-based statistical method for retrieving genes related to a query gene based on their co-occurrences in the PubMed abstracts. However, PPIFinder also employs computational linguistic methods to extract semantic descriptions of the predicted interactions from the literature. PPIFinder also searches for Gene Ontology annotations and uses the shared GO annotations to infer potential protein interactions.

According to the reported statistics [15], only 28% of the co-occurring protein pairs in PubMed abstracts appeared in any of the frequently used human PPI databases (HPRD, BioGRID and BIND). On the other hand, out of the known interacting pairs in HPRD, 69% co-occur in the literature, and 65% share GO annotations.

STRING: Search Tool for the Retrieval of Interacting Genes/Proteins [8]
The database and web-tool STRING is a meta-resource of known and predicted protein-protein associations derived from four sources of genomic context, high-throughput experiments, co-expression, and scientific literature. STRING is developed by a consortium of academic institutions, and it is regularly updated; the last version covers about 5.2 millions proteins from 1,133 species.

STRING imports protein association knowledge from databases of physical interactions and databases of curated biological pathways (e.g., MINT, HPRD, BIND, DIP, BioGRID, KEGG, and Reactome). Besides the experimentally derived gene associations, STRING also stores computationally predicted interactions from the text mining of scientific texts as well as interactions inferred from genomic features.

In terms of usage, given a query gene, STRING retrieves all genes which repeatedly occur within the same cluster as the gene of interest, where a gene cluster is defined as in [31]. Different genomic features (e.g., gene neighborhood, gene fusion events, and coexpression) are used in constructing the gene clusters. Text based predicted interactions are simply derived by searching for gene name co-occurrence in the content of PubMed abstracts [32].

4 CD82 and AMFR connectivity in the protein-protein interaction network

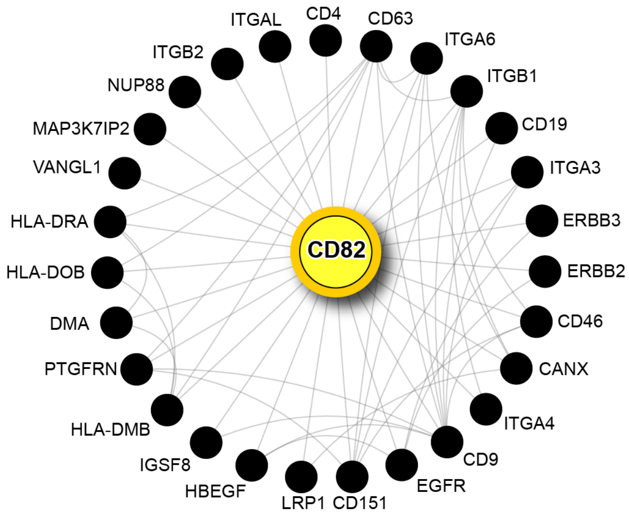

(a)

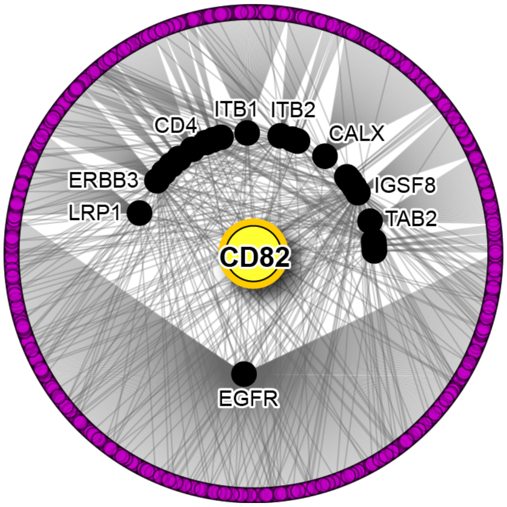

(b)

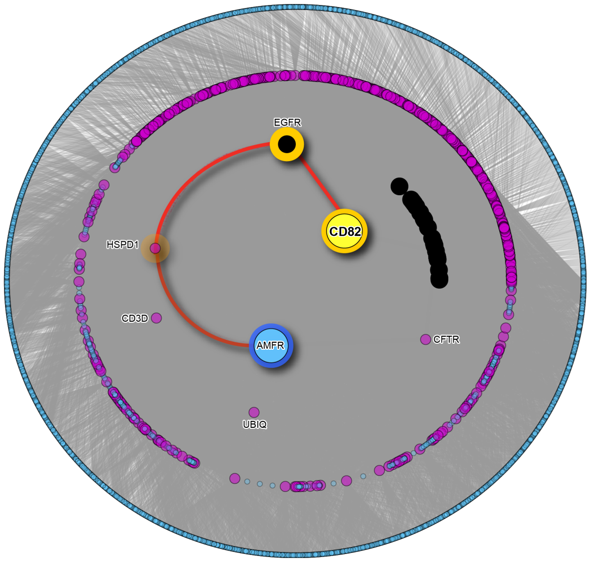

(c)

Figure S1: CD82 neighbors in the network of experimentally validated protein-protein interactions, derived from I2D-V1.95. (a): CD82's depth-1/immediate neighbors (28 genes, and 59 interactions), (b): CD82's depth-2 neighbors are added, shown by purple nodes (872 genes, and 1,209 interactions), and (c): CD82's depth-3 neighbors are added, shown by light blue nodes (7,895 genes, and 37,299 interactions). At this level, AMFR is connected to CD82 , while the network contains 60% of the nodes and 40% of the interactions of the whole PPI interactome. CD82 and AMFR are connected via ten different shortest paths of length three; one, which is via EGFR and HSPD1 is highlighted in the figure. CD82 is highly predicted by GAP to interact with AMFR, and we confirmed that this interaction is experimentally validated in the literature. Therefore, our confirmation of this GAP prediction improves the connectivity of the protein-protein interaction network. The names of genes with many connections on the next level of the network are explicitly displayed in sub-figures (b) and (c).

5 GAP's predicted functional inter-connectivity of 306 SFARI autism genes

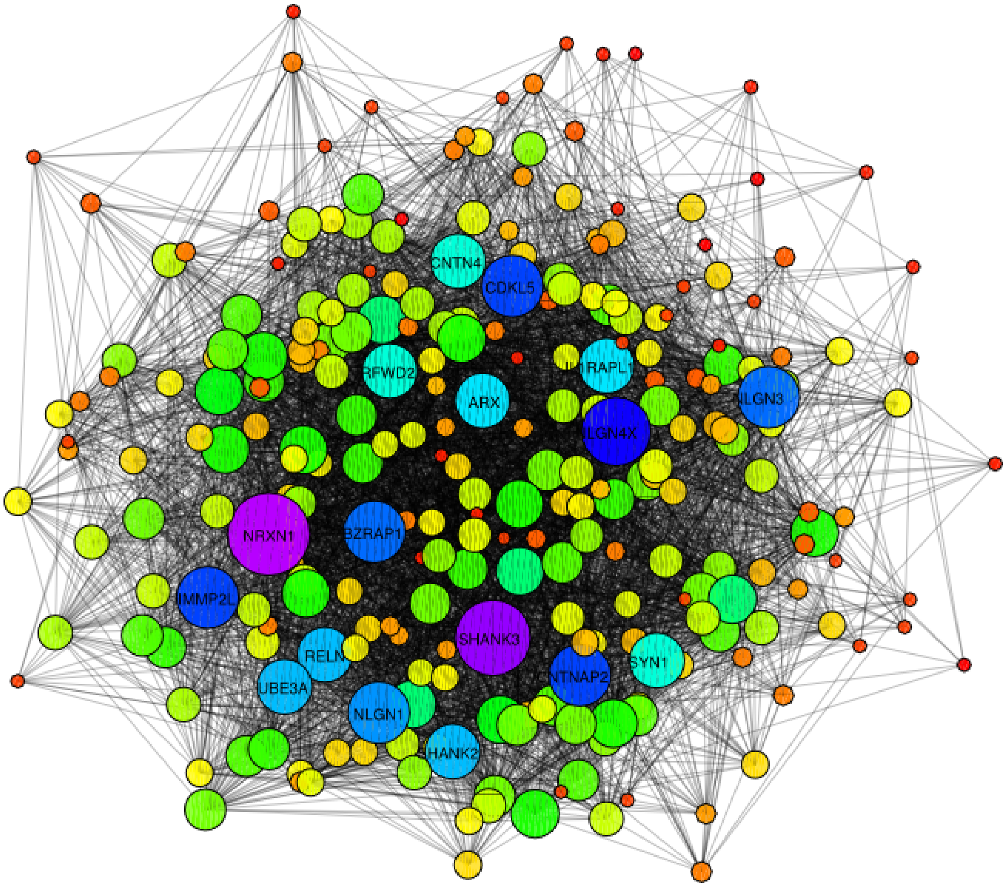

Figure S2: Functional inter-connectivity of all autism genes predicted by GAP using upper 20-quantile as threshold setting measure (p-value < 0.05). The size of each node is proportional to the node degree and the node color changes in full spectrum from red (the lowest degree) to purple (the highest degree).

Table S2: SFARI autism genes and their degree in the graph of genes' intra-associations predicted by GAP using upper 20-quantile as threshold setting measure

Gene	Degree	Gene	Degree	Gene	Degree	Gene	Degree	Gene	Degree
NRXN1	103	ATP10A	47	CTNNA3	35	ATRX	27	DCUN1D1	18
SHANK3	97	AUTS2	47	SH3KBP1	34	MAOA	27	DCTN5	17
NLGN4X	87	CADM1	47	CACNA1G	34	SYNE1	27	HRAS	17
CNTNAP2	81	SEMA5A	47	NXPH1	34	CHST5	27	AR	17
IMMP2L	80	MECP2	46	UBL7	34	SYNGAP1	27	VPS13B	17
CDKL5	79	OXTR	45	SLC30A5	34	CNTNAP5	27	BCL2	17
NLGN3	77	DLX1	45	CHD7	34	MTF1	26	VASH1	16
BZRAP1	76	MEF2C	45	SLC6A4	33	DAPK1	26	PDZD4	16
NLGN1	75	GABRA4	45	WNT2	33	PER1	26	ESRRB	16
RELN	72	LAMB1	44	CACNA1C	33	TNIP2	26	PTEN	15
UBE3A	71	MCPH1	44	FOXP1	33	ABAT	26	PCDH9	15
SHANK2	70	GRID2	44	OPRM1	33	PIK3CG	26	TBC1D4	15
IL1RAPL1	68	SLC1A1	43	HTR1B	32	GTF2I	26	TSN	14
ARX	68	ARNT2	43	SND1	32	DMPK	26	PINX1	14
RFWD2	65	RHOXF1	42	RAI1	32	PLCD1	25	TGM3	14
SYN1	64	SDC2	42	TBL1X	32	TBX1	25	FRK	14
CNTN4	64	MKL2	42	EPHA6	32	HTR2A	25	GALNT13	14
CHRNA7	63	DLX2	42	NRXN2	32	SCN2A	24	GSTM1	14
NBEA	62	ADORA2A	42	NFIA	32	ADRB2	24	CD44	13
GRIN2B	61	FOXG1	41	RPS6KA2	31	PTGS2	24	CDH22	13
HOXA1	61	UBE2H	41	CTTNBP2	31	KCNMA1	24	DHCR7	13
FBXO40	60	CDH9	41	GABRB1	31	HDAC4	24	FABP5	13
DPP6	59	GRPR	41	NIPBL	31	ITGB3	23	XPC	13
TPH2	57	CDH8	40	FBXO33	31	ESR1	23	ASS1	13
NTNG1	57	RBMS3	40	CREBBP	31	NPAS2	23	NTRK3	12
FEZF2	57	HOXB1	40	GPR139	30	PSMD10	23	HS3ST5	12
DISC1	57	CAMTA1	40	ITGB7	30	RAPGEF4	23	TH	12
CACNA1H	57	RGS7	39	GRIP1	30	KHDRBS2	23	MTHFR	12
CACNA1F	57	ASMT	39	ITGA4	30	SEZ6L2	23	ADA	12
OPHN1	55	MED12	39	RAB39B	30	MAP2	22	FGFBP3	12
APBA2	55	CACNA1D	38	DPYD	30	FHIT	22	GPX1	12
NRCAM	54	SLC9A9	37	PITX1	30	ADSL	22	GNAS	12
SLC6A8	53	BAIAP2	37	MBD3	30	AGTR2	22	RB1CC1	12
CDH10	52	AFF2	37	TDO2	30	LZTS2	22	MYO1A	11
DLX6	52	ST7	37	FABP7	30	INPP1	21	CASC4	11
PCDH19	52	MBD4	37	NRP2	30	NF1	21	HLA-A	11
DCX	51	PARK2	37	HTR3A	30	GPC6	21	TSGA14	11
NDNL2	51	KCNJ10	37	MAPK3	29	NTRK1	20	EIF4E	11
HNRNPH2	51	ARHGAP15	37	RIMS3	29	LAMC3	20	XIRP1	11
WNK3	51	HTR7	37	AFF4	29	CA6	20	ALOX5AP	10
ROBO1	50	ANKRD11	36	DRD3	29	HMGN1	20	ADK	10
TSPAN7	50	LRRC1	36	FRMPD4	29	ERBB4	20	PPP1R3F	10
DAB1	50	TLK2	36	JMJD1C	29	EGR2	20	GLO1	10
SLC25A12	50	HEPACAM	36	EML1	29	PLN	20	HSD11B1	9
SCN1A	49	HTR3C	36	RPL10	29	DPP10	19	HLA-DRB1	9
NLGN4Y	49	STK39	36	DMD	28	PECR	19	TTN	9
EN2	49	DIAPH3	36	RBFOX1	28	DDX11	19	CBS	7
FGD1	49	NSD1	36	MACROD2	28	SLC4A10	19	BTAF1	7
FOXP2	48	PCDH10	36	ATP2B2	28	APC	18	NDUFA5	7
MDGA2	48	ASTN2	36	MBD1	28	AHI1	18	C4B	6
REEP3	48	LRFN5	35	ALDH5A1	28	FLT1	18	TAF1L	5
IL1RAPL2	47	SLC9A6	35	SUCLG2	28	F13A1	18		
MARK1	47	SCFD2	35	FMR1	28	PON1	18		
DLGAP2	47	NOS1AP	35	ICA1	27	TSC2	18		

6 Autism-related genes predicted by GAP while novel to the SFARI database

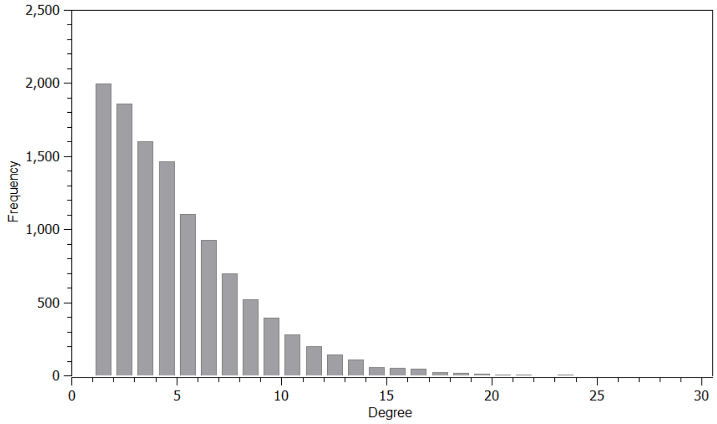

Figure S3: Histogram of the association-degrees of 11,215 genes predicted to be functionally associated to SFARI known autism genes. Association-degree of a predicted gene corresponds to the number of SFARI autism genes predicted to be functionally associated to it.

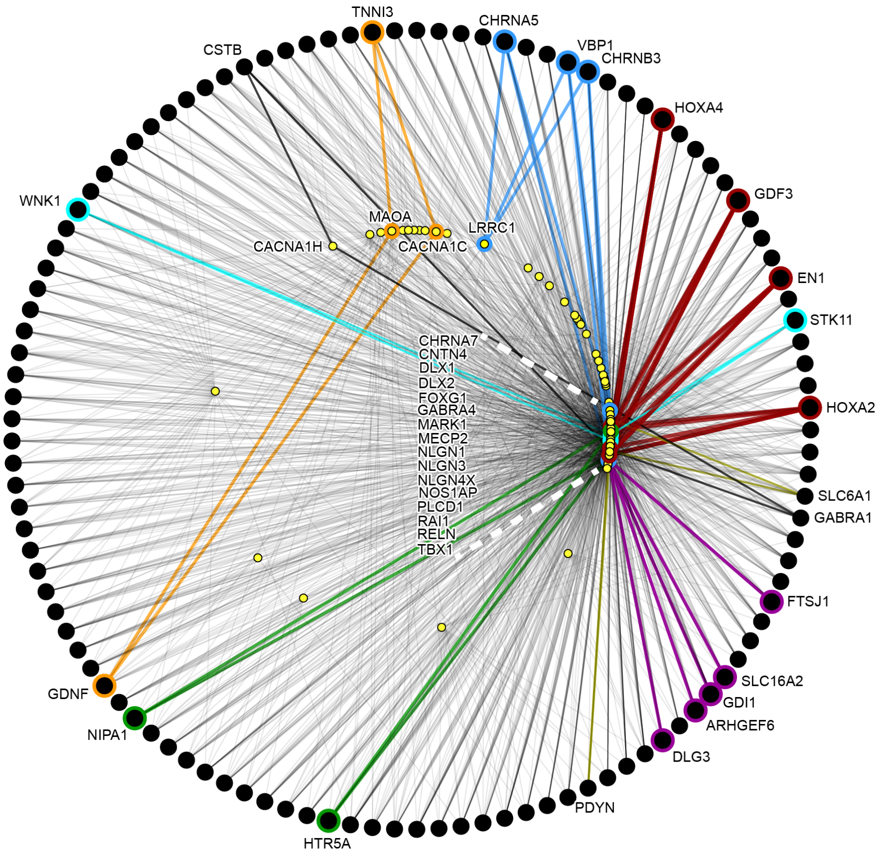

(a)

Sub-graph	Nodes	Edges	Density	Min-degree	Max-degree	Ave-degree
Cluster1	9	20	0.56	14	39	19.56
Cluster2	6	9	0.60	17	19	17.50
Cluster3	7	10	0.48	15	35	20.0
Cluster4	4	4	0.67	12	24	18.25
Cluster5	4	4	0.67	8	18	14.00
Cluster6	4	4	0.67	19	25	22.25
Cluster7	4	4	0.67	13	16	14.50
Cluster8	4	4	0.67	7	23	13.50

(b)

Figure S4: (a): Network of functional associations among SFARI known autism genes and novel autism genes predicted by GAP. Yellow nodes are SFARI genes, and black nodes are 114 novel autism genes. Edges correspond to predicted functional associations between novel and known genes (edges among SFARI autism genes are filtered out). The predicted novel genes are densely connected to autism genes, and form a highly-interconnected network with several clique-like subgraphs as highlighted in the graph. Approximate cliques are identified using NAViGaTOR's [33] plug-in to the NeAT toolbox [34]. (b):Information of the clique-like subgraphs highlighted in sub-figure (a). Second and third columns of the table give the total number of nodes and edges of each sub-graph, respectively. *Density* refers the proportion of the edges in the corresponding complete graph covered by the sub-graph, i.e., $Density = Edges \Big/ \binom{Node}{2}$. The last three columns display for each sub-graph, the minimum, the maximum, and the average values of the node degrees, respectively.

7 GAP's predicted functional interactome

GAP's high confidence ($p-text-value < 0.01$; estimated by a phenotype-based permutation test) predicted "functional interactome" contains ≈1M functional associations among about 19K human genes. Out of these, about 90% are novel (i.e., not listed in publicly available datasets of experimentally validated direct and indirect interaction). GAP's novel predictions connect previously disconnected components and singletons to the main body of the known interactome and are shown in Figure S5.

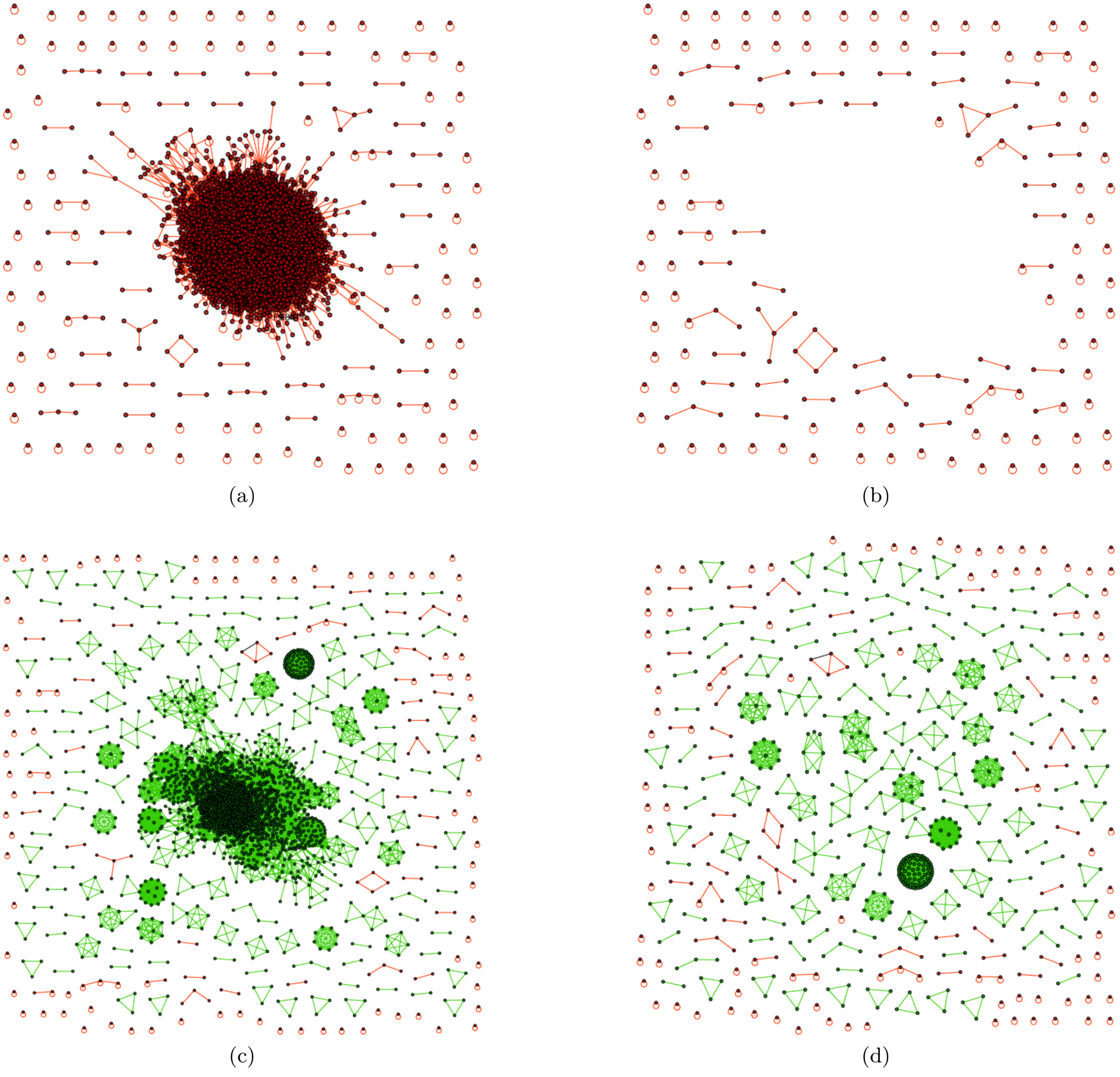

Figure S5: (a): Network of experimentally validated protein-protein interactions used as positive gold-standard database in our study. (b): Disconnected components and singletons in the network of sub-figure (a). (c): Network of sub-figure (b) plus the network of human experimentally known co-complex associations used as positive gold-standard database in our study. (d): Disconnected components and singletons in the network of sub-figure (c).

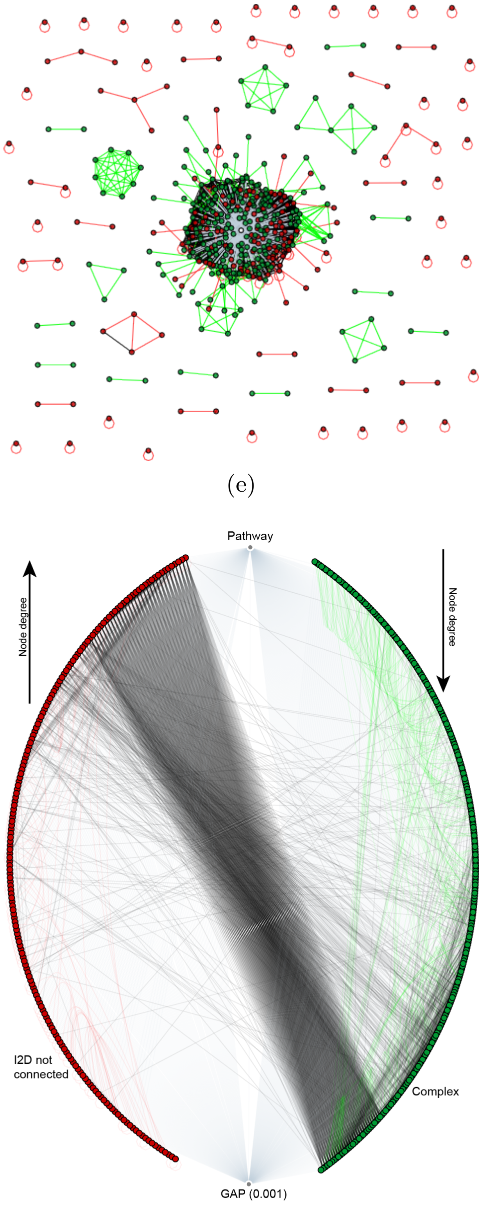

(e)

(f)

Figure S5 (Cont.): (e): Network of sub-figure (d) plus the network of human experimentally known co-pathway memberships used as positive gold-standard database in our study. Some of the disconnected components and singletons in network (d) are connected through the pathway functional associations. Nodes not included in sub-figure (d), but associated to at least one of the genes in sub-figure (d) are shown as a single collapsed-node in the center of this figure. (f): Network of sub-figure (e) plus GAP's predicted novel associations whose p-value is less than 0.001. Predicted associations among genes which are not included in the network of sub-figure (e) are excluded in order to reduce the complexity for visualizing the functional interactome (the goal here is to show how predicted interactions are relating disconnected components of known interactome). Nodes not included in sub-figure (d), but associated to at least one of the genes in sub-figure (d) are shown as a single collapsed-node at the bottom of the sub-figure; the collapsed node at the top of the sub-figure is identical to that of sub-figure (e). Network of sub-figure (f) has 203,622 edges from which about 50% are GAP's predicted novel associations.

References

[1] Resnik P: **Using information content to evaluate semantic similarity in a taxonomy**. *Int Joint Conf for AI (IJCAI-95)* 1995, :448–453.

[2] Seco N, et al.: **An intrinsic information content metric for semantic similarity in WordNet**. *16th European Conf on AI* 2004.

[3] Jones K: *Information retrieval experiment.* London: Butterworths.

[4] Raghavan V, et al.: **A critical investigation of recall and precision as measures of retrieval system performance**. *ACM Transactions on Information Systems (TOIS)* 1989, **7**(3):205–229.

[5] Fawcett T: **ROC graphs: notes and practical considerations for data mining researchers** 2003. [Technical Report HPL-2003-4, HP Labs].

[6] Flach P, Matsubara ET: **Obtaining calibrated probability estimates from simple lexicographic rankings**. *In the 18th European Conference on Machine Learning ECML* 2007, :575–582.

[7] Hanley JA, McNeil BJ: **The meaning and use of the area under a receiver operating characteristic (ROC) curve**. *Radiology* 1982, **143**(11):29–36.

[8] Szklarczyk D, et al.: **The STRING database in 2011: functional interaction networks of proteins, globally integrated and scored**. *Nucleic Acids Res* 2011, **39**:D561–8.

[9] Tuncbag N, et al.: **Predicting protein-protein interactions on a proteome scale by matching structural similarities at the interface using PRISM**. *Nature Protocols* 2011.

[10] Chang DT, et al.: **Predicting the protein-protein interactions using primary structures with predicted protein surface**. *BMC Bioinformatics* 2010, **11**:S3.

[11] Mohamed TP, et al.: **Active learning for human protein-protein interaction prediction**. *BMC Bioinformatics* 2010, **11**:S57.

[12] Lee I, et al.: **Predicting genetic modifier loci using functional gene networks**. *Genome Research* 2010, **20**(8):1143–1153.

[13] Singhal M, et al.: **Using support vector machine for improving protein-protein interaction prediction utilizing domain interactions**. *Proceedings of the First ACM International Conference on Bioinformatics and Computational Biology* 2010, **10:36**:537–545.

[14] Wu G, et al.: **A human functional protein interaction network and its application to cancer data analysis**. *Genome Biology* 2010, **11:R53**.

[15] He M, et al.: **PPI Finder: a mining tool for human protein-protein interactions**. *PLoS ONE* 2009, **4**(2):e4554.

[16] McDowall M, et al.: **PIPs: Human protein-protein interactions prediction database**. *Nucleic Acids Res* 2009, **37**:651–656.

[17] Park SH, et al.: **Prediction of protein-protein interaction types using association rule based classification**. *BMC Bioinformatics* 2009, **10:36**.

[18] Rao R, et al.: **Amino-acid residue association models for large scale protein-protein interaction prediction**. *In Silico Biology* 2009, **9**(4):179–194.

[19] Guo Y, et al.: **Using support vector machine combined with auto covariance to predict protein-protein interactions from protein sequences**. *Nucleic Acids Res.* 2008, **36**:3025–3030.

[20] Mostafavi S, et al.: **GeneMANIA: a real-time multiple association network integration algorithm for predicting gene function**. *Genome Biol.* 2008, **9**:S4.

[21] Shen J, et al.: **Predicting protein-protein interactions based only on sequences information**. *PNAS* 2007, **14**(11):4337–4341.

[22] Pall F, et al.: **Cluster analysis of networks generated through homology: automatic identification of important protein communities involved in cancer metastasis**. *BMC Bioinformatics* 2006, **7**(2).

[23] Ben-Hur A, Noble WS: **Kernel methods for predicting protein-protein interactions**. *Bioinformatics* 2005, **21**:i3846.

[24] Brown K, Jurisica I: **Unequal evolutionary conservation of human protein interactions in interologous networks**. *Genome Biology* 2007, **8**(5):R95.

[25] Chen X, Liu M: **Prediction of protein-protein interactions using random decision forest framework**. *Bioinformatics* 2005, **21**(24):4394–4400.

[26] Han D, et al.: **PreSPI: a domain combination based prediction system for protein-protein interaction**. *Nucl. Acids Res.* 2004, **32**(21):6312–6320.

[27] Aloy PH, Russell RB: **InterPreTS: protein interaction prediction through tertiary structure**. *Bioinformatics* 2003, **19**:161–162.

[28] Bock JR, Gough DA: **Predicting protein-protein interactions from primary structure**. *Bioinformatics* 2001, **17**(5):45560.

[29] Salwinski L, et al.: **The database of interacting proteins: 2004 update**. *Nucleic Acids Res* 2004, **32**(Database issue):D449–51.

[30] Pagel P, et al.: **The MIPS mammalian protein-protein interaction database**. *Bioinformatics* 2005, **21**(6):832–4.

[31] Overbeek R, et al.: **The use of gene clusters to infer functional coupling**. *Proc. Natl Acad. Sci, USA* 1999, **96**:2896–2901.

[32] von Merin C, et al.: **STRING: a database of predicted functional associations between proteins**. *Nucleic Acids Res.* 2003, **31**:258–61.

[33] Djebbari A, et al.: **NAViGaTOR: scalable and interactive navigation and analysis of large graphs**. *Internet Mathematics* 2011, **7**(4):314–347.

[34] Brohee S, et al.: **NeAT: a toolbox for the analysis of biological networks, clusters, classes and pathways**. *Nucleic Acids Res* 2008, **36**(Web Server issue):W444451.
